# Patterns of benthic bacterial diversity in coastal areas contaminated by heavy metals, polycyclic aromatic hydrocarbons (PAHs) and polychlorinated biphenyls (PCBs)

**DOI:** 10.3389/fmicb.2015.01053

**Published:** 2015-10-13

**Authors:** Grazia Marina Quero, Daniele Cassin, Margherita Botter, Laura Perini, Gian Marco Luna

**Affiliations:** National Research Council-Institute of Marine Sciences (CNR-ISMAR), VeneziaItaly

**Keywords:** microbial diversity, chemical pollution, PCBs, marine sediments, next generation sequencing

## Abstract

Prokaryotes in coastal sediments are fundamental players in the ecosystem functioning and regulate processes relevant in the global biogeochemical cycles. Nevertheless, knowledge on benthic microbial diversity patterns across spatial scales, or as function to anthropogenic influence, is still limited. We investigated the microbial diversity in two of the most chemically polluted sites along the coast of Italy. One site is the Po River Prodelta (Northern Adriatic Sea), which receives contaminant discharge from one of the largest rivers in Europe. The other site, the Mar Piccolo of Taranto (Ionian Sea), is a chronically polluted area due to steel production plants, oil refineries, and intense maritime traffic. We collected sediments from 30 stations along gradients of contamination, and studied prokaryotic diversity using Illumina sequencing of amplicons of a 16S rDNA gene fragment. The main sediment variables and the concentration of eleven metals, polychlorinated biphenyls (PCBs) and polycyclic aromatic hydrocarbons (PAHs) were measured. Chemical analyses confirmed the high contamination in both sites, with concentrations of PCBs particularly high and often exceeding the sediment guidelines. The analysis of more than 3 millions 16S rDNA sequences showed that richness decreased with higher contamination levels. Multivariate analyses showed that contaminants significantly shaped community composition. Assemblages differed significantly between the two sites, but showed wide within-site variations related with spatial gradients in the chemical contamination, and the presence of a core set of OTUs shared by the two geographically distant sites. A larger importance of PCB-degrading taxa was observed in the Mar Piccolo, suggesting their potential selection in this historically polluted site. Our results indicate that sediment contamination by multiple contaminants significantly alter benthic prokaryotic diversity in coastal areas, and suggests considering the potential contribution of the resident microbes to contaminant bioremediation actions.

## Introduction

Coastal marine ecosystems are amongst the most productive and diverse on Earth, providing over US$ 14 trillion worth of ecosystem goods (Harley et al., 2006). The human impact is altering the coastal sea functioning under the consequence of a plethora of pressures, including chemical pollution and wastewater discharge, eutrophication, hypoxia, utilization of living resources (e.g., over-fishing), habitat destruction, and climate change effects. The diversity and functioning of coastal ecosystems are largely affected from chemical pollution by a plethora of compounds, including those identified as emergent (Elliott and Elliott, 2013). Contaminants accumulate in marine and freshwater environments, and determine the reduction of biodiversity, by means of adverse effects to the resident biota, the removal of sensitive species and the selection of the more tolerant ones (Johnston and Roberts, 2009). The human impact in the coastal ocean is becoming evident also at the microbial level (Paerl et al., 2003), with the more obvious effects in terms of inputs of autochthonous and pathogenic microbes (spreading diseases to human and marine populations; Stewart et al., 2008), shifts in community composition, and impairment of ecological functions (Nogales et al., 2011). Marine microbes are extremely sensitive to environmental changes because of their small size, fast growth rates and genome plasticity. Consequently, the study of microbial community diversity, and their fluctuations over spatial and temporal scales, represents a useful tool to evaluate the consequences of the anthropogenic perturbation on marine ecosystem health (Ager et al., 2010).

Among chemical contaminants which are recovered in the coastal environment, heavy metals, polychlorinated biphenyls (PCBs) and polycyclic aromatic hydrocarbons (PAHs) represent some of the most ubiquitous and spread (Förstner and Wittmann, 1983; Fernandes et al., 1997; Field and Sierra-Alvarez, 2008), and occur primarily as a result of anthropogenic inputs. Heavy metals are highly persistent and may exert toxic effects at all levels of biological organization, from cells to population, and community structure, by altering enzymatic functioning and metabolic pathways (Chapman et al., 1998). PCBs are stable organic molecules that have been largely used, for more than 50 years, for a wide number of industrial applications because of their convenient physical and chemical properties. Once in the environment, PCBs accumulate along the food web and can exert multiple adverse effects for marine life and human health, even under low environmental concentrations (Borja et al., 2005; Carpenter, 2006). Finally, PAHs are aromatic compounds produced mainly during combustion in natural and anthropogenic processes, and are typically found in high concentrations on industrial sites, particularly those associated with the petroleum, gas-production, and wood-preserving industries (Wilson and Jones, 1993). They cause concern as environmental pollutants because some are carcinogens and mutagens, and have a high potential for biomagnification through the aquatic food web due to their lipophilic nature. All these types of contaminants accumulate in coastal marine sediments representing their main depository, where they can exert a significant influence on the benthic biota (Rocchetti et al., 2012).

A number of studies have been performed to shed light on the influence of chemical contaminants on microbial communities in coastal marine sediments. High concentrations of heavy metals have been shown to inhibit benthic bacterial metabolism and turnover (Dell’Anno et al., 2003), as well to influence the diversity of resident microbes and to select for those more tolerant to metals (Gillan et al., 2005). Nonetheless, certain aquatic microbes play a role into the fate and cycling of toxic metals (Ford and Ryan, 1995), as well in the biodegradation of PCBs (Bedard, 2008; Field and Sierra-Alvarez, 2008; Pieper and Seeger, 2008), and a variety of bacteria are capable of transforming and mineralizing PAHs (Kanaly and Harayama, 2000). Studies to investigate relationships between contaminants and bacteria reported lower diversity and richness in polluted sediments, and dominance of few OTUs (Sun et al., 2012), likely resulting from selective advantage of those microbes able to metabolize contaminants. Jonhston and Leff (2015) reported that bacterial community composition was strongly influenced by PAHs in riverbank sediments. The large majority of studies in coastal sediments have been performed with the aim of isolating and characterizing pollutant-degrading bacteria, and to evaluate the potential for sediment remediation. Conversely, only few studies addressed the potentially combined effects of multiple contaminants, such as heavy metals, PAHs and PCBs together, on the diversity and spatial patterns of complex sediment communities (Sun et al., 2012; Brito et al., 2015), and even less studies have used to this purpose the recently developed next generation sequencing (NGS) techniques (Sun et al., 2013; Korlević et al., 2015), which allow detailed characterizations of the rare and dominant taxa within assemblages. Consequently, our study has potential to widen significantly our limited understanding of the bacterial community response to sediment pollution in coastal areas.

The Po River Prodelta (Northern Adriatic Sea) and the Mar Piccolo of Taranto (Ionian Sea) are two contaminated sites, located in the northern and southern coast of Italy, respectively. The Po River Prodelta receives significant contaminant discharge from one of the largest rivers in Europe (Boldrin et al., 2005). The delta system has a daily mean discharge of 1500 m^3^/s (ranging from 100 m^3^/s to 11550 m^3^/s) and produces a freshwater plume able to influence the whole Adriatic Sea (Falcieri et al., 2014), including the functioning of sediment microbes (Manini et al., 2004). The Po typically experiences major floods, during which transport and depositional processes lead large amounts of suspended sediments and associated contaminants, which can be stored or transported offshore to the adjacent areas (Correggiari et al., 2005). Many studies have shown that this site is severely chemically polluted, as the river carries yearly tons of anthropogenic chemicals collected from the entire Po valley and the river tributaries (Viganò et al., 2003). The Mar Piccolo of Taranto (Ionian Sea) is an inner, semi-enclosed basin which communicates with the adjacent Ionian Sea through two channels (Petronio et al., 2012). It is a chronically polluted area due to the presence, since decades, of the largest steel production plant in Europe. It also hosts a variety of other sources of pollutants, among which oil refineries, a large naval base (including a military ship-yard) and intense maritime traffic, and has received in the past considerable amounts of sewage from several pipes discharges (Cardellicchio et al., 1997). This chronical contamination is now evident in terms of PCBs, PAHs, and heavy metal concentrations in the sediments (Cardellicchio et al., 1997; Petronio et al., 2012). Knowledge on the biodiversity of benthic bacteria in the two study sites is today scant or non-existent, being limited only to the Mar Piccolo site where cultivation-based studies have described fecal bacteria and the biodiversity of culturable microbes only (Cavallo et al., 1999; Zaccone et al., 2005). There is currently no information available obtained using the recent NGS techniques, consequently our understanding of the microbial processes and the potential of resident microbes for remediation actions is largely hampered.

In this study, we collected sediments from 30 stations, located along gradients of putative sediment contamination, in the two sites and we described in detail, by NGS of the 16S rRNA gene, the bacterial richness, diversity, and community composition. Diversity was studied as a function of the main environmental variables and the concentration of eleven heavy metals, PCBs and PAHs, to test the hypothesis that the chemical contamination influences the richness and community composition of benthic prokaryotes. This study is among the first to investigate, by mean of a large sequencing effort (consisting of millions of 16S rDNA sequences), the combined effects of multiple contaminants on benthic prokaryotes across different spatial scales. Moreover, hypotheses on the potential contribution of the resident microbes to contaminant bioremediation actions are given.

## Materials and Methods

### Sampling Sites and Activities

**FIGURE 1:**
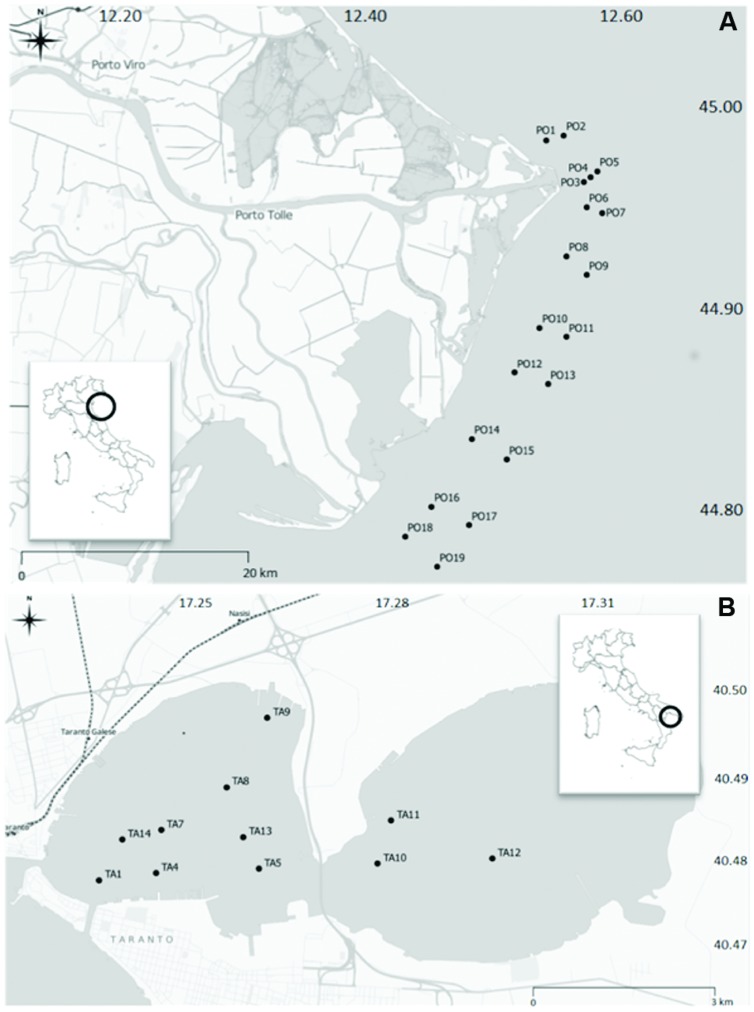
**The study sites.** The two sites sampled in Italy: the Po River Prodelta **(A)** and the Mar Piccolo of Taranto **(B)**.

Surface sediments were collected in two sites located on the eastern coast of Italy: the Po River Prodelta (Northern Adriatic Sea) and the Mar Piccolo of Taranto (Ionian Sea). Sampling activities were performed in the periods between 10th and 14th June 2013 in the Po River Prodelta, and between 17th and 21th June in the Mar Piccolo of Taranto. The experimental design in the Po River Prodelta site included 19 stations, which were distributed along coast to open sea transects (**Figure 1A**), at depths comprised between 9–21 mt. The sampling transects were placed in front of the outlets (Busa di Tramontana, Busa Dritta, Busa di Scirocco) of the main branches of the river delta (Po di Maistra, Po della Pila, Po di Tolle, Po della Gnocca, Po di Goro). The distribution of stations was chosen in order to follow the possible deposition of material transported in the area following an exceptional flood event occurred in the third week of May 2013 with a maximum of flow rate measured in Pontelagoscuro (FE) equal to 6830 m^3^/sec. The Mar Piccolo of Taranto (**Figure 1B**) displays a restricted circulation and extends for a total surface area of 20.7 km^2^. It is structured into two inlets, the “First Inlet” (having the maximum depth of 13 meters) and the “Second Inlet” (maximum depth 8 meters; Cardellicchio et al., 2007). The Mar Piccolo is connected with the adjacent Mar Grande through two channels, termed “Navigabile” and “Porta Napoli”. In terms of the hydrographic characteristics, this site can be compared to a brackish lake. Salinity is influenced by the input of freshwater originating by small tributary rivers and freshwater springs called “Citri” (Cavallo et al., 1999). The sampling design in this area included 11 stations, eight of which located in the First and three in the Second inlet, at depths comprised between 6 and 12 mt. One station (TA1) was located immediately in front of the channel linking the Mar Piccolo with the adjacent open sea. Basing on CTD measurements in the water column (full data are not shown), the Po River Prodelta showed a more marked influence of the river flow regime on the hydrological characteristics, evident in the wide range of temperature (16.4–24.9°C) and salinity values (6.10–37.56 PSU). Conversely, the Mar Piccolo of Taranto showed more stable hydrological conditions, with temperature and salinity values in the range 21.0–23.5°C and 36.0–38.4 PSU (respectively), highlighting poor fresh water inputs during the sampling period. Detailed geographical coordinates and sampling depths of all stations in the two sites are reported in the Supplementary Tables S1 and S2. Samples were collected in triplicate using a Van Veen grab sampler (capacity 10 L) onboard small research vessels. Only in the site of Taranto, the sediments for chemical analysis were collected with a gravity corer (model SW-104). Once onboard, the uppermost 0–2 cm layer of each sediment sample was immediately processed according to the specific protocols required for each type of analysis. For the analysis of organic pollutants an aliquot of the sediment was put in a hexane-rinsed aluminum foil and stored at -20° C until analysis. For heavy metals, organic matter content and grain size determinations, the sediments were put in a PET jar and stored at 4°C until analysis. With regard to the sediment cores, they were kept vertical at the temperature of 4°C until being processed into different sub-samples. For microbiological analyses, the sediment was immediately placed, using sterile spatulas, under sterile containers for their immediate transport at 4°C to the laboratory, where the samples were then stored at -20°C until molecular analyses of biodiversity.

### Environmental Variables and Chemical Contaminants

The organic matter content was estimated by the method based on loss on ignition (LOI) at 550°C for 2 h. The grain-size distribution was measured by means of a laser beam analyser (Microtrac mod. X-100, Leeds and Northrup, USA). For metals analysis, each sediment sample was gently squeezed to break down aggregates, screened through a sieve with a mesh of 1 mm and the dried sediment was ground to power using an agate mortar. The sample (about 0.4 g d.w.) was then digested with 8 ml HNO_3_ in a microwave oven (Multiwave 3000, Anton Paar, Austria). The digested was left to cool at room temperature and then filtered through a 0.45 μm nitrocellulose membrane filter. The filtered digestates were diluted with distilled deionised water to 40 ml in a volumetric flask (U.S. Environment Protection Agency of United States of America, 1994a). The concentrations of the metals (Al, As, Cd, Cr, Cu, Fe, Mn, Ni, Pb, and Zn) and total P content were determined by inductively coupled plasma atomic emission spectrometry (ICP-AES) (Optima 2100DV, Perkin Elmer, USA; U.S. Environment Protection Agency of United States of America, 1994b). Mercury analyses were carried out by atomic absorption spectrophotometry by cold vapor (Analyst 100, Perkin Elmer, USA; U.S. Environment Protection Agency of United States of America, 1976). The precision of instrumental analysis was checked by control chart of each heavy metal. Data quality was monitored using 10% procedure blanks and 10% sample replicates. Recovery, checked analyzing a certified reference material for heavy metals (BCR-277r estuarine sediment, Community Bureau of Reference). For organic pollutants, samples were air dried in the dark at room temperature for 48 h on hexane-rinsed aluminum foil and then the dry samples were finely ground in a agate mortar. The extraction of about 2 g of sediment was performed using a Microwave Sample Preparation System, in accordance with the EPA recommendation (U.S. Environment Protection Agency of United States of America, 2007) with a 25 ml 1:1 acetone/hexane solvent mixture. The samples were concentrated in a rotating evaporator (Rotavapor-R Buchi, CH), and the sulfur compounds were removed by soaking the extracts with activated copper powder. Purification and fractionation were performed by eluting extracts through chromatography glass columns packed with Silica gel/Alumina/Florisil (4 + 4 + 1 g). The first fraction, containing PCBs, was eluted with 25 ml of n-hexane, whereas the second fraction, containing the PAHs, was eluted with 30 ml of 8:2 n-hexane/methylene chloride solvent mixture (Fossato et al., 1996, 1998). The concentration of 16 USEPA priority pollutant PAHs were analyzed with high performance liquid chromatograph (PE 200, USA), coupled to a programmed fluorescence detector (HP 1046A, USA). The column used was a reverse-phase Supelcosil LC-PAH (*L* = 150 mm, *f* = 3 cm, 5 μm). PCBs (32 congeners) were analyzed by gas chromatography/mass spectrometry (GC/MS). The system consist of an Agilent 7820A GC coupled with an Agilent 5977E Series GC/MS, and the software MassHunter for data analysis. The GC is equipped with a 30 m HP-5MS capillary column (0.25 mm ID, 0.25 μm film). The identification of PAHs and PCBs was based on matching retention time, and the quantification was determined from calibration curves established for each compound by analyzing five external standards. The method detection limits, measured using the calibration curve method, ranged between 0.05 and 0.1 ng g^-1^ for PAHs, and 0.05 ng g^-1^ for PCBs. Blanks were run for the entire procedure. Validation of the recovery and accuracy was carried out with IAEA-417 and IAEA-159 sediment sample certified reference materials.

### Bacterial Diversity Analyses using Illumina Sequencing

DNA was extracted from 1 g of each sediment sample using the PowerSoil^®^ DNA Isolation Kit (MoBio Laboratories Inc., California), according to the manufacturer’s instructions with some slight modifications to increase the DNA yield and quality. These modifications included two additional vortexing steps (following the one which is recommended by the manufacturer) at the maximum speed for 2 min, each one being preceded by an incubation at 70°C for 5 min, and by adding one more washing step with Solution C5 as an additional removal step for contaminants. The concentration of each DNA extract was determined spectrophotometrically, and the DNA was stored at -80°C until PCR. Illumina Miseq V3 sequencing were carried out on the hypervariable V3 and V4 regions of the 16S rRNA gene by amplifying using the 341F (5′-CCTACGGGNGGCWGCAG-3′) and 785R (5′-GACTACHVGGGTATCTAATCC-3′) universal bacterial primers (Eiler et al., 2012). Paired-end reads were quality checked (with default settings and minimum quality score of 20) and analyzed with QIIME v1.8.0 software package (Quantitative Insights Into Microbial Ecology) (Caporaso et al., 2010). Reads were clustered into OTUs by using UCLUST v1.2.22 (Edgar, 2010) with a >97% similarity threshold with a open-reference OTU picking strategy and default settings. Chimeras were detected by using USEARCH v6.1 (Edgar, 2010). Chimera checking and taxonomy assignment was performed using Greengenes 13.8 as reference database (De Santis et al., 2006). Abundances in each sample were normalized on the number of sequences of sample with the lowest number of reads retained. The sequences have been submitted to the SRA -Sequence Read Archive (accession number SRP061637).

### Data Handling and Statistical Analyses

The distribution maps which show the concentrations of some contaminants were produced as a contour plot based on geographic information system (GIS) technology. The software used was QGIS and the interpolation was carried out with inverse distance weighted method (IDW) with power parameter equal to 2. The Spearman-Rank correlation analysis was performed to test linear relationships between some of the microbiological variables and the concentration of environmental and chemical contaminants. Correlation coefficients (*r*) were considered significant at *p*-values less than 0.05. Differences in the community composition between the two sites were assessed, on the Illumina dataset, using the analysis of similarity (ANOSIM) tool based on a Bray–Curtis similarity matrix. The presence of statistical differences between samples is indicated by a significance level at *p*-values less than 0.05. Similarity was calculated by performing an UPGMA clustering based on unweighted Unifrac distance matrix (Lozupone and Knight, 2005). A PCOa (Principal Coordinates analysis) was performed on the environmental variables and chemical contaminants to explore and visualize similarities among the two sites. This analysis was performed basing on normalized data and using a Euclidean distance matrix. Multivariate, multiple regression analyses were performed to identify drivers of bacterial community composition in the investigated samples. The analysis was performed using the Distance-based linear modeling (DistLM) analysis on the Illumina-based resemblance matrix (Bray–Curtis similarity) at either phylum and OTUs level and including the main environmental variables (total P content, % LOI, and silt) and chemical contaminants (Al, As, Cd, Cr, Cu, Fe, Hg, Mn, Ni, PAHs, Pb, PCBs, and Zn, or a selection of them as later specified) as predictor variables. Prior of each DistLM analysis, the values of environmental variables and chemical contaminants were normalized. We used, as selection procedure, the option “All specified,” and AICc as the selection criterion. A distance-based redundancy analysis (dbRDA) plot was prepared using all the tested variables. The ANOSIM, PCOa, DistLM, and dbRDA analyses were performed using the PRIMER 6 + software (http://www.primer-e.com/).

## Results

### Analysis of Environmental Variables and Contaminants

**FIGURE 2:**
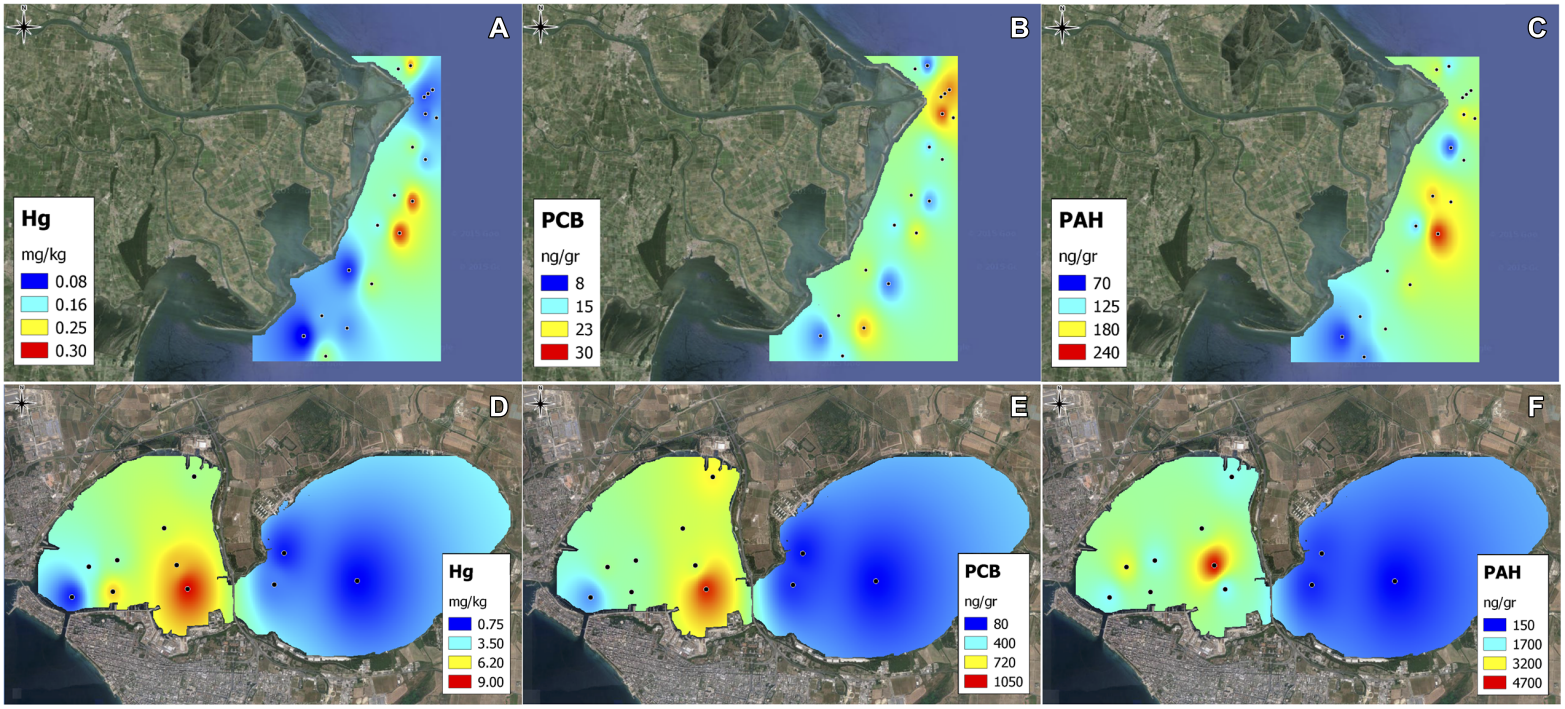
**Spatial patterns of contaminants.** Spatial patterns of selected chemical contaminants in the two sites. Shown are mercury **(A,D)**, polychlorinated biphenyls (PCBs) **(B,E)** and polycyclic aromatic hydrocarbons (PAHs) **(C,F)** in the Po River Prodelta and the Mar Piccolo of Taranto, respectively.

The concentration of all the environmental variables and chemical contaminants in the two sites is reported in Supplementary Table S1 (Po River Prodelta) and Supplementary Table S2 (Mar Piccolo), while the spatial distribution of selected contaminants in the two sites is shown in **Figures 2A–F.** Furthermore, the concentration of the measured PAHs and PCBs congeners is reported in Supplementary Tables S3 and S4. Surface sediments appeared as mainly constituted by fine materials, but differences were observed between the two sites. In the Po River Prodelta, the percentage of silt, clay, and sand showed an average of 68, 18, and 14%, respectively. The silty fraction showed a very low variability (coefficient of variation CV = 6.2%), while the sandy component showed a wider range (CV = 52%). In this site, a decreasing trend in the sandy percentage from north to south was observed. Conversely, in the Mar Piccolo of Taranto, the sandy fraction was on average twice compared with the other site. Silt and clay accounted for 56 and 15%, respectively. More uniform textural features were evident, with CV of 18, 6, and 16% for sand, silt and clay, respectively. The station TA1 differed from all other stations, with a very high percentage of sand (79%) and a very low silt (17%) and clay (<5%) content. The analysis of chemical contaminants highlighted different levels of contamination in the two sites. The Po River Prodelta site showed an overall lower contamination level. Mercury concentration displayed very low concentrations, always below the current Italian regulatory limits (0.3 mg kg^-1^ according to DL 260/2010). Chromium and nickel showed high concentrations (94 and 77 mg kg^-1^, respectively). PCBs concentrations always exceeded the same regulatory limits (which are set to 8 ng g^-1^). PAHs showed overall low concentrations (148 ng gr^-1^ on average), well below the abovementioned limits (set to 800 ng gr^-1^), with the highest values observed at the stations PO10, PO11, and PO13. Among the measured PAHs congeners, fluoranthene and pyrene were those displaying, as average of all stations, the higher concentration. Among the PCBs congeners, the dominant were 138, 153, and 180. The Mar Piccolo of Taranto site showed much higher concentrations of contaminants, especially in terms of arsenic, copper, lead, zinc, mercury, PAHs, and PCBs. For the last three contaminants, the values resulted to be on average 24, 12, and 25 times higher, respectively, than in the Po River Prodelta. The dominant PAHs congeners were pyrene and benzo(g,h,j)perylene + indeno(1,2,3-cd)pyrene. Chemical analyses demonstrated the presence of a very large number of PCBs congeners. The most contaminated area was the one located in front of the arsenal of the Italian Navy (e.g., TA5), which showed very high concentration of several contaminants and particularly mercury and PCBs (9.0 mg kg^-1^ and 1045 ng g^-1^, respectively). Chromium and nickel showed low values, while Fe, Al, Cd, and total P showed values comparable to those found in the other study site.

The Principal Coordinates analysis of the environmental variables and chemical contaminants showed a clear separation between the two sites (Supplementary Figure S1). This separation was evident also considering the only group of Mar Piccolo of Taranto stations, in which two separate subgroups can be identified according to the location of the stations in the first and second inlet. The station TA1 differed completely from all other stations, which reflects its peculiar textural characteristics (very high percentage of sand).

### Bacterial Diversity, Community Composition, and Core Microbiome

A cumulative number of 3,500,760 raw sequences was obtained for the 30 stations from the Illumina sequencing analyses. The average value was 116,692 reads per sample, with a minimum number of 30,119 in the station PO4 and the maximum number of 201,629 in the station PO10. The average length of sequences in the entire dataset was 418.9 ± 1.35 base pairs. After the quality check, the final dataset included 2,953,054 reads, with an average value of 98,453 per sample (minimum number of 25,354 at station PO4 and maximum number of 168,785 at station PO10), with an average length of 418.5 ± 12.41 base pairs.

Bacterial OTU richness in the two study sites ranged from 3,992 (PO1) to 7,640 (PO7) OTUs in the Po River Prodelta, and from 1,103 (TA12) to 6,752 (TA1) OTUs in the Mar Piccolo of Taranto (Supplementary Figure S2). Typically, richness displayed higher values in the Po River Prodelta (on average 5,898 OTUs) than in the Mar Piccolo samples (on average 3,809 OTUs).

**FIGURE 3:**
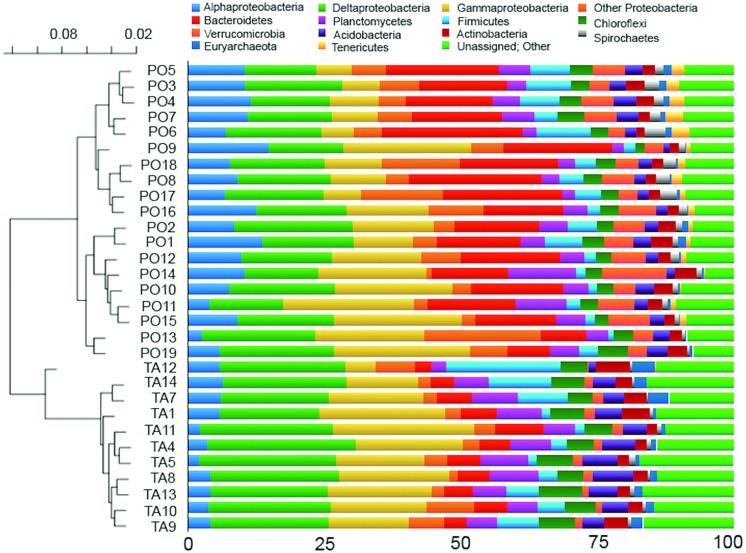
**Community composition and Unweighted Pair Group Method with Arithmetic mean (UPGMA) dendrogram.** Combined panel showing the relative abundance of prokaryotic phyla, or classes (for Proteobacteria), in the sampled stations **(Right)** and the UPGMA dendrogram, based on unweighted Unifrac distances matrix **(Left)**. On the right panel, phyla or classes showing a mean relative abundance across all stations <1%, as well as all the unassigned sequences, were aggregated into the group reported as “Unassigned; Other.”

Bacterial community composition showed that OTUs were affiliated to 81 known and 2 unknown phyla, and to 284 known and 64 unknown classes. The relative importance of the most abundant phyla and classes is shown in **Figure 3** (right). In all samples, the phylum Proteobacteria represented the most abundant (on average 47.6 and 48.6% in Po River Prodelta and Mar Piccolo, respectively). Within this phylum, the classes Delta- (average 16.9 and 22.6%, respectively) and Gammaproteobacteria (average 14.6 and 17.4%, respectively) were the two most frequently observed. Alphaproteobacteria accounted for 9.1 and 4.4% (respectively), while the other Proteobacteria (including Beta-, Epsilon-, Zeta-, and TA18) accounted for 6.9 and 4.1%, respectively. The second most abundant phylum was Bacteroidetes (average 26% in Po River Prodelta and 9% in Mar Piccolo). Among the other most dominant phyla, Planctomycetes accounted for 2.1–12.3 and 2.5–9% (Po River Prodelta and Mar Piccolo, respectively), followed in importance by Firmicutes (range 0.9–10% in Po River Prodelta, and 1.5–20.9% in Mar Piccolo), and Chloroflexi (1.6–5.5% in Po River Prodelta, 4.5–7.9% in Mar Piccolo). Other phyla included Verrucomicrobia (average 5.4 and 1.5% in the two sites, respectively), Acidobacteria (average 2.7 and 4.8%, respectively), Actinobacteria (2.6 and 3.3%, respectively) and Spirochaetes (1.6 and 0.6%, respectively). Despite the primers we used are designed to target mostly Bacteria and do not provide a representative picture of the whole archaeal assemblages, it is worth mentioning that we found a low frequency of Euryarchaeota (0.7 and 1.8% respectively), especially in the Mar Piccolo samples. Finally, Tenericutes were detected infrequently (average value 1.3 and 0.04% in Po River Prodelta and Mar Piccolo, respectively). The percentage of “Unassigned and Other sequences” was 2.4% in the Prodelta River Po and 3.3% in the Mar Piccolo. The UPGMA clustering based on unweighted Unifrac distance revealed a clear separation between the two study areas (**Figure 3**, left). ANOSIM confirmed the presence of significant differences in community composition at the phylum level between the two sites (*r* = 0.704, *p* < 0.01).

The analyses at the OTU level indicated that the cumulative number of OTUs observed in the two sites was 56,917 OTUs, with a total number of 43,613 OTUs recorded in the Po River Prodelta, and the total number of 20,235 OTUs in the Mar Piccolo of Taranto. In the Po River Prodelta site, the most abundant OTUs (here defined as those accounting for at least >1% across all stations within the each site) were represented by one OTU belonging to the Helicobacteraceae family (class Epsilonproteobacteria) which displayed the average percentage of 3.05% of the total reads. This OTU was followed in importance by two OTUs within the Deltaproteobacteria class, the first belonging to the family Desulfuromonadaceae (average 1.62%) and the second to the family Desulfobulbaceae (1.15%), by a gammaproteobacterial OTUs in the family Piscirickettsiaceae (1.08%) and lastly by another OTU affiliated to the genus Lutimonas within the Bacteroidetes phylum (average 1.06%). In the Mar Piccolo site, the most abundant OTUs were one identified as belonging to the genus Thermoanaerobacter (phylum Firmicutes, average 2.96%), followed by one OTU in the Syntrophobacteraceae family (Deltaproteobacteria, 2.28%), the same OTUs observed in the Po River Prodelta site within the Helicobacteraceae family (1.59%) and the family Desulfobulbaceae (1.05%), and one OTU in the genus Tissierella/Soehngenia (Firmicutes, 1.20%). These dominant OTUs represented altogether 7.96 and 9.08% of the total OTU abundance in the two sites, respectively. The remaining less abundant OTUs, contributing each for <1%, represented the largest fraction of the obtained sequences within each station and site (92.04 and 90.92% of the sequences in the two sites, respectively).

**FIGURE 4:**
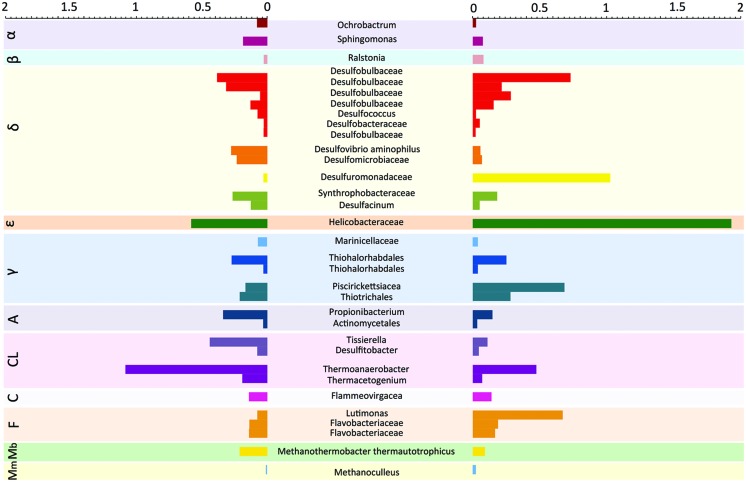
**Core microbiome in contaminated coastal sediments.** Relative abundance (expressed as %) of OTUs shared between the Po River Prodelta **(Left)** and Mar Piccolo of Taranto **(Right)** sites. The OTU identity is reported the highest level of taxonomic identification obtained using QIIME (which ranged from Family to Species). Letters in the colored boxes in background describe the Classes to which each OTUs are affiliated (α = Alphaproteobacteria; β = Betaproteobacteria, δ = Deltaproteobacteria, 𝜀 = Epsilobacteria, γ = Gammaproteobacteria, A = Actinobacteria, Cl = Clostridia, C = Cytophagia, F = Flavobacteria, Mb = Methanobacteria, Mm = Methanomicrobia).

When the sites were compared at the OTU level, we found a core microbiome which comprised 33 OTUs, 31 of which belonged to Bacteria and 2 to Archaea (**Figure 4**). These OTUs were observed consistently at all stations of the two sites with at least one read. This core microbiome comprised some of the same OTUs previously identified as dominant, such as the same OTU within the Helicobacteraceae family observed at all stations, and the OTU belonging to the genus Thermoanaerobacter which was particularly abundant in the Mar Piccolo site. The remaining core microbiome OTUs were spread across 11 bacterial and archaeal classes.

### Relationships between Contaminants and Community Composition

**FIGURE 5:**
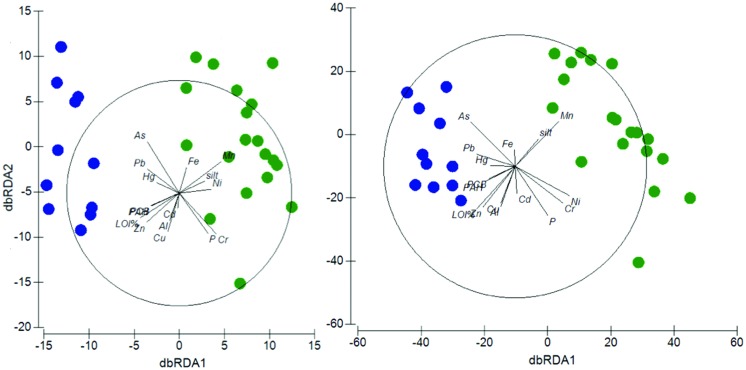
**Relationships between environmental variables, contaminants and taxonomic composition across sites.** dbRDA ordinations of the distLM model which describe the relationship between the environmental variables (P, % LOI, silt content), the chemical contaminants (Al, As, Cd, Cr, Cu, Fe, Hg, Mn, Ni, PAHs, Pb, PCBs, and Zn) and the taxonomic composition at the phylum **(Left)** and OTU **(Right)** level. In the phylum plot, the first axis (dbRDA1) captures 57.6% of the fitted and 42.3% of the total variation between the samples’ taxonomic profile at the phylum level, while the second (dbRDA2) captures 26.3% of the fitted and 19.2% of the total variation. In the OTU plot, the first axis (dbRDA1) captures 47% of the fitted and 33.9% of the total variation between the samples’ taxonomic profile at the OTU level, while the second (dbRDA2) captures 14.3% of the fitted and 10.3% of the total variation. Green circles: Po River Prodelta samples. Blue circles: Mar Piccolo of Taranto samples.

OTU richness typically showed a decreasing pattern with increasing chemical contamination in the sediment. This pattern was reflected in the significant negative correlations observed between richness and the concentration of several contaminants, among which PCBs (*r* = 0.439, *p* < 0.05), PAHs (*r* = 0.439, *p* < 0.05), Hg (*r* = 0.397, *p* < 0.05), Cu (*r* = 0.507, *p* < 0.001), Pb (*r* = 0.487, *p* < 0.01), As (*r* = 0.408, *p* < 0.05), Zn (*r* = 0.616, *p* < 0.001), and Al (*r* = 0.397, *p* < 0.05). DistLM analyses performed using all the environmental and chemical variables, except for sand and clay (which showed redundancy with the “silt” variable and were thus not included in the analyses) showed that, at the phylum level, all the considered variables, with the only exception of Al, Cd, and Fe, were able to significantly explain the differences observed in the community composition at p < 0.001 significance (Supplementary Table S5), while total P explained significantly but at lower significance level (p < 0.05). A similar output of the distLM was observed at the OTU level (Supplementary Table S6). The cumulative percentage of variance explained by the set of environmental and chemical variables was 73.3 and 72.1% at the phylum and OTU level, respectively. A dbRDA analysis was used for the graphical visualization of the DistLM results. The dbRDA plot showed that, at both phylum and OTU level, the environmental and chemical parameters divided the stations into two separate clusters corresponding to the two study sites (**Figure 5**). The two axes of the phylum- and OTU-dbRDA explained 61.5 and 44.2% of the total variation, respectively.

**FIGURE 6:**
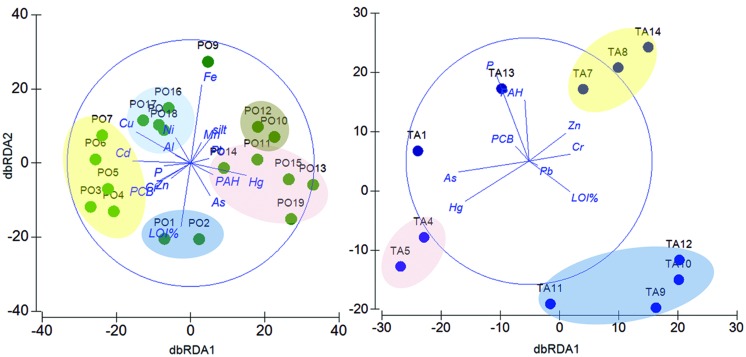
**Relationships between environmental variables, contaminants and taxonomic composition within each site.** dbRDA ordinations of the distLM model which describe the relationship between the environmental variables (P, % LOI, silt content), the chemical contaminants (Al, As, Cd, Cr, Cu, Fe, Hg, Mn, Ni, PAHs, Pb, PCBs, and Zn) and the taxonomic composition at the OTU level in the Po River Prodelta **(Left)** and Mar Piccolo of Taranto **(Right)** sites. In the Po River Prodelta plot, the first axis (dbRDA1) captures 19.1% of the fitted and 17.1% of the total variation between the samples’ taxonomic profile, while the second (dbRDA2) captures 8.1% of the fitted and 7.2% of the total variation. In the Mar Piccolo of Taranto plot, the first axis (dbRDA1) captures 22.4% of the fitted and 18.1% of the total variation between the samples’ taxonomic profile, while the second (dbRDA2) captures 20% of the fitted and 16.2% of the total variation. Green circles: Po River Prodelta samples. Blue circles: Mar Piccolo of Taranto samples.

According to the evident clustering between the two sites, we performed separate DistLM and dbRDA analyses at the OTU level to test the role of environmental variables and chemical contaminants in shaping community composition within each sampling site. In the Po River Prodelta, the DistLM analysis indicated a significant influence of several environmental variables and chemical contaminants, which together explained up to 89.8% of community variation. The dbRDA plot showed that the environmental and chemical parameters structured the stations into several separate clusters (**Figure 6**, left). These included groups of stations located along gradients characterized by different level of contamination, among which one group made up by the stations PO1 and PO2 (which were best correlated to % LOI, *r* = -0.514), a second one containing the stations from PO3 to PO7, which were best structured by Cu (*r* = -0.445), Cd (*r* = 0.472), and PCB (*r* = -0.587), a third group containing the stations PO8, PO16, PO17, and PO18, best related to Cu (*r* = -0.445), Cr (*r* = -0.251), and Al (*r* = -0.123; Supplementary Table S7). The plot showed an outlier station which was mostly related to Fe (PO9, *r* = 0.632), another group including two stations (PO10 and PO12) which were best related to Mn (*r* = 0.167) and silt (*r* = 0.203) and finally a large cluster comprising the remaining five stations, mostly structured by Hg (*r* = 0.453), As (*r* = -0.262), and PAHs (*r* = 0.184).

In the Mar Piccolo site, the DistLM analysis similarly showed a significant influence for certain environmental variables and contaminants. The dbRDA plot divided the stations into three major groups (**Figure 6**, right), one containing the stations TA9, TA10, TA11 and TA12 (which were best correlated to % LOI, *r* = -0.466), another one including TA7, TA8, and TA14 (best correlated with Zn, *r* = 0.301) and a third group of stations including TA4 and TA5 (best related with Hg, *r* = -0.518; Supplementary Table S8). The remaining stations appear to be scattered on the plot and differently influenced by other variables.

## Discussion

Coastal marine sediments represent biogeochemically relevant areas of the global ocean, where prokaryotes are recognized to play a significant role (Luna et al., 2002; Gobet et al., 2012). Chemical pollutants, such as heavy metals and xenobiotics, can accumulate in sediments in highly anthropized areas, and potentially influence prokaryotic communities. However, relatively few studies have investigated bacterial diversity and community composition in highly polluted sediments (Gillan et al., 2005; Paissé et al., 2008; Zhang et al., 2008; Sun et al., 2012), and even less using the NGS techniques (Sun et al., 2013; Korlević et al., 2015). This study was performed to shed light on the role of chemical pollutants in shaping bacterial diversity in coastal contaminated sediments. The study was carried out in two contrasting areas along the coastline of Italy, characterized by different type and level of chemical contaminants. The chemical analyses confirmed this difference, evident in terms of higher concentration of Ni, Cr, and Mn in the Po River Prodelta site, and in much higher concentrations (up to one hundred times) of certain pollutants, among which the more relevant were Hg, PAHs, and PCBs, in the chronically polluted Mar Piccolo site. These results corroborate previous studies carried out in these areas to investigate spatio-temporal patterns of contaminants (Cardellicchio et al., 2007; Petronio et al., 2012). In the Po River Prodelta site, Cr and Ni showed a high concentration, probably due to the enrichment by the leaching of sedimentary ophiolite complexes, which emerge in the Western Alps and some areas of the Italian Apennine. The elevated Cr and Ni backgrounds are therefore a geogenic character of the Po River alluvial sediments and likely unrelated to anthropogenic contamination (Bianchini et al., 2013). Conversely, the influence of the plume on the enrichment of PCBs in sediments was reflected in the highest values observed in front of the main river mouths. In the Mar Piccolo of Taranto, the most contaminated area was located in front of the arsenal of the Italian Navy, which showed the highest concentrations of several pollutants of concern and, particularly, Hg and PCBs. An additional source of PCBs contamination was found in the vicinity of shipyards in the north of the first shelf of Mar Piccolo. On the other hand, Cr and Ni showed very low values, consistent with the background values reported in the area (Buccolieri et al., 2006).

### Patterns of Richness and Community Composition in Contaminated Coastal Sediments

OTU richness showed a decreasing pattern with increasing chemical contamination in the sediment. A negative correlation between richness and contaminants has been previously reported in soils (Joynt et al., 2006; Gołębiewski et al., 2014), while contrasting patterns have been reported in marine sediments. A pattern similar to our has been observed in South–Eastern Australia in metal-and PAH-contaminated sediments (Sun et al., 2012) as well as in certain, despite not at all sites, contaminated sediments in the Northern Adriatic Sea (Korlević et al., 2015). A similar decrease in richness was observed when benthic bacterial communities from a contaminated harbor were exposed to hydrocarbons in laboratory experiments (Yakimov et al., 2005). Conversely, other studies, performed using a community fingerprinting technique, highlighted that OTU richness was unaffected by the sediment contamination (Gillan et al., 2005), while Zhang et al. (2008) reported higher richness in the reference site with respect to the more polluted ones. The lack of consistent patterns in richness can be explained by a multitude of reasons, assuming that the response of complex communities to chemical contamination may vary according to the magnitude-dependent toxic effect of pollutants (Ager et al., 2010; Sun et al., 2012), but also that contaminants may favor, over time scales, the proliferation of microbial consortia of more tolerant species replacing non-tolerant ones, leading in turn to increases in diversity (Gillan et al., 2005). Also, the use of different microbiological techniques to describe richness (e.g., fingerprinting, cloning and NGS of the 16S rRNA gene) can lead to different results, as fingerprinting methods describe only the richness of dominant species while NGS allows identifying dominant and rare species within the communities (Bent et al., 2007). Our results, among the first based on large sets (millions) of 16S rDNA sequences, indicate that chemical contamination reduce benthic prokaryotic richness in coastal sediments. This pattern agrees with ecological theories predicting that multiple stressors lead to diversity decreases, because of inability of certain individuals to develop tolerance (Vinebrooke et al., 2004).

Despite the negative correlation between richness and contaminants, bacterial community composition showed a very high taxonomic diversity at both sites, with OTUs affiliated to more than 80 phyla and more than 300 classes. This taxonomic composition appears wider than reported by Sun et al. (2013) and Korlević et al. (2015), and this can also be related with the use of different sequencing techniques, with our use of the Illumina platform leading to higher number of detected taxa (Wang et al., 2012). We show that community composition in both sites was dominated by Delta- and Gammaproteobacteria, followed in importance by Alphaproteobacteria. This pattern is similar to that described by Sun et al. (2013). Conversely to our findings, Korlević et al. (2015) reported dominance of Gamma- and Alphaproteobacteria, and an overall lower frequency of Deltaproteobacteria, which were mostly observed in the more PAH-contaminated station. The dominance of the classes is not unexpected, as Gamma- and Alphaproteobacteria are widely recognized to utilize aliphatic and aromatic compounds, and to play a key role in oil degradation (Kostka et al., 2011; Acosta-González et al., 2013), and members of Deltaproteobacteria are crucially involved in the anaerobic degradation of organic contaminants and the cycling of sulfur compounds (Gillan et al., 2005; Sun et al., 2013). In our samples, the percentage of Deltaproteobacteria appeared to be significantly and positively correlated to the concentration of PAHs (at all sites, *p* < 0.05, *r* = 0.517) suggesting the presence of a tight link between contaminants and the growth of members of this class.

The second most abundant phylum in our samples was Bacteroidetes. This is in accordance with previous studies in contaminated sediments (Paissé et al., 2008; Sun et al., 2013; Korlević et al., 2015). This class appeared particularly important in the Po River Prodelta, where their percentage is up to three times higher than in previous studies. Members of the Bacteroidetes phylum are known to represent one of the most abundant groups of bacteria in coastal areas, and can be found as free-living or associated with organic particles (Fernández-Gómez et al., 2013). The high relevance observed in contaminated sites, and particularly in the estuarine one, can be explained by considering their potential freshwater origin, and the known ability of members of this phylum to tolerate the toxic effects of certain metals (Oregaard and Sørensen, 2007), or alternatively to grow on some contaminants, such as hydrocarbons, as substrates (Yakimov et al., 2007). Firmicutes, Chloroflexi, and Planctomycetes were also abundant at both sites, followed by less represented phyla such as Verrucomicrobia, Acidobacteria, Actinobacteria, and Spirochaetes. All these phyla have been widely reported as members of contaminated sediments communities (Dell’Anno et al., 2012), and this can be associated to their role as heavy metals-reducers and polyaromatic compounds- and PCB-degraders, or to their ability to survive to toxic effects of metals (Nogales et al., 2001; Petrie et al., 2003; Zhang et al., 2008; Zanaroli et al., 2012).

It is worth noting that all our samples contained, despite at low percentages, members of the class Epsilonproteobacteria. This class included one dominant OTU (belonging to the Helicobacteraceae family) which was consistently identified among the most abundant in all sediment samples. A similar finding was reported by Korlević et al. (2015), who showed that this taxon, along with the Lachnospiraceae family, accounted for 60% of the total sequences. At the same time, the dominance of Helicobacteraceae has been observed in sediments collected from the Arctic Mid-Ocean ridge (Jorgensen et al., 2012), where the sulfur cycle was found to be particularly relevant. The dominance of this OTU within Helicobacteraceae in contaminated coastal sediments deserves further investigation, as this class includes members able to survive to high toxic effects (Mitchell et al., 2014) and, given their known involvement in the sulfur cycle, suggests that this process may be particularly relevant in contaminated coastal sites. The other dominant OTUs were affiliated with the sulfur-reducing Desulfuromonadaceae family (Deltaproteobacteria), whose members have been previously found in contaminated sediments (Gillan et al., 2005; Sun et al., 2013), as well within the family Desulfobulbaceae, observed in artificial oil-spill and harbor sediments (Zhang et al., 2008; Suárez-Suárez et al., 2011). Other dominant OTUs belonged to the sulfur-oxidizing Piscirickettsiaceae family and to the genus Lutimonas. These taxa have been reported among the most abundant during laboratory-scale experiments of dechlorination of contaminated sediments (Zanaroli et al., 2012; Koo et al., 2014), suggesting their possible decontamination role in heavily polluted areas. In the Mar Piccolo site, the dominant OTU belonged to phylum Firmicutes, whose members have been already reported in similar environments worldwide and include several hydrocarbon-degrading species (Zhang et al., 2008; Korlević et al., 2015).

### A Core OTUs Microbiome in Contaminated Sediments

We found a core sediment microbiome comprising 31 OTUs belonging to Bacteria and 2 belonging to Archaea, which were consistently recorded at all stations of the study sites. Sun et al. (2013) reported a core sediment microbiome made up of 13 OTUs in a study carried out in six polluted estuaries in SE Australia. However, these authors reported that the core OTUs were mostly comprised of Gamma-, Delta-, Alphaproteobacteria, and Acidobacteria. Our results, in contrast, show a more diversified microbiome, which also includes OTUs belonging to Beta- and Epsilonbacteria, Clostridia, Cytophagia, Flavobacteria, and to Archaea (within the classes Methanobacteria and Methanomicrobia). These differences can be related to the geographic distance, as well to the different type, level and history of pollution between the sites targeted in the two studies. For instance, the sites investigated by Sun et al. (2013) showed, on average, three times lower concentration of Cu, six times lower concentration of Ni and two times lower concentration of Pb, as well as differences in the type and concentration of PAHs congeners (such as presence of naphthalene and acenaphthylene, which were absent in our sites). Nonetheless, both studies point out, for the first time, that highly polluted coastal environments, despite being located at very long distances (more than eight hundreds kilometers in our case), does appear to share common microbial players.

Given that non-polluted sites were not analyzed in our study, we cannot exclude that these core OTUs may be present in the area independently of the contamination. However, in partial support of our hypothesis, we observed that several core OTUs displayed lower importance in those stations displaying the lower contamination, which suggests that contamination may have promoted the growth and selection of these OTUs. This appears true, in the Po River Prodelta, for several OTUs (among which Thermoanaerobacter, several Deltaproteobacteria and Methanomicrobia) in the station presenting the lowest level of contamination (PO10). The same was observed in the Mar Piccolo of Taranto, where more than half of the core OTUs were less abundant (up to 28 times) in the less contaminated station (TA1) than in the other ones. Future studies will have to provide support to the existence of a core microbiome in contaminated areas, and to elucidate the role that this core set of microbes plays in the ecosystem functioning and biogeochemical cycles in coastal areas under anthropogenic stress.

### Gradients and Type of Contaminants Shape Bacterial Diversity and Community Composition

The distLM analyses showed that the environmental and chemical variables, with the only exclusion of some metals (Al, Cd, and Fe), significantly shaped community composition at the two sites, by explaining a large fraction of observed variance (73.3 and 72.1% at phylum and OTU level, respectively). Our results corroborates previous findings reporting significant effects of contaminants in driving sediment bacterial community composition (Sun et al., 2012, 2013; Jonhston and Leff, 2015). Sun et al. (2012) reported a significant role of metals and PAHs in driving changes in bacterial communities. Paissé et al. (2008) showed that 32% of variation in bacterial communities along a hydrocarbon contamination gradient was significant explained by PAHs. The fraction of explained variance in our study appears relatively high compared with previous studies, suggesting that contaminants and environmental variables are playing a significant role in structuring communities in the investigated sites. However, the remaining fraction of unexplained variance is likely to depend upon other factors which were not investigated in this study, such as biotic factors (predation, viral lysis and completion), deserving future investigations.

The further dbRDA plot showed that the environmental and chemical parameters divided the stations into two separate clusters corresponding to the study sites, suggesting the existence of important within-site differences. These differences are likely dependent upon a different role played by the geographical and environmental characteristics on communities, and upon the presence of spatial gradients in the concentration and type of pollutants. In the Po River Prodelta, the environmental and chemical parameters structured the 19 stations into several clusters, which corresponded to groups of stations located along gradients of pollution, and characterized by different contamination level. Stations PO1 and PO2 were located northern of the delta main distributary mouth (Po della Pila), in an area under minor influence of river discharge, as river waters discharged to the region are typically flowing south (Boldrin et al., 2005). This finding is confirmed by the average concentration of certain contaminants (such as Ni, Mn, Al, and PCBs) which appears lower in these two stations than in remaining ones located southward. Accordingly, the second cluster of stations (from PO3 to PO7), which was located in front or south of the main distributary mouth, were the most contaminated in terms of more than half of the measured contaminants, and particularly by PCBs (on average 25 ng g^-1^). Thus, communities in this cluster of station are likely to have been shaped together by the higher contaminant pressure, resulting also from the recent flood event which preceded our sampling. The remaining clusters of stations in this site appeared to be structured differently according to contaminant levels, type of anthropogenic influence and distance from land and from the other tributary mouths.

In the Mar Piccolo site, the environmental and chemical parameters structured the 11 stations into three major clusters and some outlier stations. One outlier was TA1, which is located right in front of the “Navigabile” channel connecting the Mar Piccolo with the adjacent open sea. This station was characterized by different environmental characteristics (higher % of sand and lower organic matter content), lower contaminant level (evident especially in terms of metals and PCBs) and a more marked influence of the marine waters entering into the inlet. This influence is likely the reason for the differences in community composition between this station and the other ones, evident for instance in terms of lower importance of Firmicutes and higher contribution of other taxa, such as Vibrionales (which were up to seven times more abundant at this station). The other major clusters of stations were also related with different characteristics. One cluster comprised stations TA4 and TA5, which were located in the nearby of the arsenal of the Italian Navy, and displayed the highest concentration of Hg, As, and PCBs. Station TA13 clustered apart, being characterized by the highest concentrations of PAHs and of total P of the entire study area. Another cluster included TA7, TA8, and TA14, which were located in the middle of the First Inlet, and likely received a similar combined effect of multiple contaminants (including PCBs) which may have shaped similar communities. A remaining cluster grouped together all those stations located in the Second Inlet. This Inlet is less polluted than the First in terms of most of the analyzed contaminants. In this cluster, the station TA12 showed an increased relevance of Clostridia (phylum Firmicutes), accounting for more than 20% of the sequences. Within this class, two of the five dominant OTUs observed in the entire study site (Thermoanaerobacter and Tissierella/Soehngenia) reached percentages up to 3 and 12 times (respectively) higher than the average values in the other stations. The high relevance of Clostridia in this station may be related to the presence of dense mussel culture facilities, which supports previous results in the same study area stressing the role of these bacteria as bioindicator of aquaculture impact (Zaccone et al., 2005).

The two sites here studied also differed significantly in terms of PCBs concentration (ANOVA, *p* < 0.01), with the Mar Piccolo site showing the average concentration of 466.72 ng g^-1^ (standard error 89.29 ng g^-1^), which was up to 25 times higher than in the Po River Prodelta (average 18.71 ng g^-1^, standard error 1.19 ng g^-1^). This striking difference was reflected in the microbial community composition, and in the increased relevance of certain taxa which are well known as PCB-degraders. The largest difference was observed in terms of Chloroflexi, which were on average twofold more abundant in Mar Piccolo than the Po River Prodelta. PCBs are known to be degraded by several prokaryotes under both aerobic and anaerobic types of metabolism (Furukawa and Fujihara, 2008; Pieper and Seeger, 2008). PCB-degrading bacteria span a variety of taxonomic groups, among which the gram negative genera *Pseudomonas*, *Alcaligenes*, *Achromobacter*, *Burkholderia*, *Comamonas*, *Sphingomonas*, *Ralstonia*, and *Acinetobacter*, and the Gram-positive genera *Rhodococcus*, *Corynebacterium*, and *Bacillus* (Field and Sierra-Alvarez, 2008). Enrichment cultures experiments indicate that several *Dehalococcoides* sp. and other microorganisms within the Chloroflexi phylum can grow by linking the oxidation of H_2_ to the reductive dechlorination of PCBs (Fagervold et al., 2007). The percentage of Chloroflexi in our samples was significantly and positively related with the concentration of PCBs (*r* = 0.42, *p* < 0.05) suggesting the potential selection of PCB-degrading communities in the more polluted stations. This hypothesis was further corroborated by the presence of significant correlations between Chloroflexi and certain PCBs congeners, such as 99, 110, and 126 (*r* = 0.44, *r* = 0.43, and *r* = 0.61, *p* < 0.05, respectively). It is clear that future studies should use more accurate quantification techniques, such as quantitative PCR targeting specific genes (the 16S rRNA gene of *Dehalococcoides*), to quantify the abundance of degrading populations, and confirm the significance of these relationships. At the same time, future researches are needed to prove the functional capacity of these populations and to identify their active fraction, basing on the analysis of the 16S rRNA rather than 16S rDNA or other approaches to quantify functions (Blazewicz et al., 2013). Chlorinated biphenyls are potentially fully biodegradable, a process which is made possible in a sequence of anaerobic reductive dechlorination reactions, followed by aerobic mineralization of the lower chlorinated products (Field and Sierra-Alvarez, 2008). Our results suggest the possible role of certain autochthonous populations as key agents in sediment PCBs degradation processes, and as potential targets for future remediation actions to improve the environmental quality of the investigated sites, and to design and test on-site bioremediation actions by the sediment naturally dechlorinating microorganisms, as recently demonstrated in laboratory experiments in the Mar Piccolo site (Matturro et al., 2015).

## Conclusion

We conclude that sediment contamination by multiple chemicals do significantly shape benthic prokaryotic diversity in coastal areas, and suggest considering the potential contribution of resident microbes to actions of contaminant remediation. While our results add new knowledge about the anthropogenic influence on coastal habitats and the response of benthic microbes, future studies will need to elucidate how and whether multiple contaminants influence the functioning of the sediment microbiome, to identify microbial indicator taxa for human alteration, and to evaluate the ecological and biogeochemical consequences of chemical pollution on coastal ecosystems.

## Conflict of Interest Statement

The authors declare that the research was conducted in the absence of any commercial or financial relationships that could be construed as a potential conflict of interest.
