# Supplementary material for: Patterns of benthic bacterial diversity in coastal areas contaminated by heavy metals, polycyclic aromatic hydrocarbons (PAHs) and polychlorinated biphenyls (PCBs)
Source: Front Microbiol. 2015 Oct 13;6:1053. doi: 10.3389/fmicb.2015.01053 (PMC4602156; doi:10.3389/fmicb.2015.01053)

***Supplementary Material***

**Patterns of benthic bacterial diversity in coastal areas contaminated by heavy metals, Polycyclic Aromatic Hydrocarbons (PAHs) and Polychlorinated Biphenyls (PCBs)**

**Grazia Marina Quero, Daniele Cassin, Margherita Botter, Laura Perini, Gian Marco Luna***

Institute of Marine Sciences (CNR-ISMAR), National Research Council, Venezia, Italy

*** Correspondence:** Gian Marco Luna, National Research Council, Institute of Marine Sciences (CNR-ISMAR), Castello 2737/f, Arsenale Tesa 104, 30122 Venezia, Italy. Tel.: +39 041 2407993 email: gianmarco.luna@ve.ismar.cnr.it

**Supplementary Table 1, 2, 3, 4, 5, and 6.**

**Supplementary Figure 1 and 2.**

**Supplementary Table 1.** Position (as latitude and longitude) of the sampling stations within the site Po River Prodelta and concentration of heavy metals (as mg Kg^-1^), organic matter content (expressed as % of LOI, loss on ignition), PAHs and PCBs (as ng gr^-1^) and grain size (expressed as % of sands, silt and clay) in the sediments.

|  |  |  |  |  |  |  |  |  |  |  |  |  |  |  |  |  |  |  |  |  |  |  |  |  |
| --- | --- | --- | --- | --- | --- | --- | --- | --- | --- | --- | --- | --- | --- | --- | --- | --- | --- | --- | --- | --- | --- | --- | --- | --- |
|  | **N** | **E** | **mt** | **mg Kg^-1^** | | | | | | | | | | |  | **ng gr^-1^** | |  | **mg Kg^-1^** | **%** |  | **%** | | |
| **Station** | **Lat** | **Long** | **Depth** | **Hg** | **Cr** | **Cu** | **Ni** | **Pb** | **As** | **Cd** | **Zn** | **Mn** | **Fe** | **Al** |  | **PAHs** | **PCBs** |  | **P** | **LOI** |  | **Sand** | **Silt** | **Clay** |
| **PO1** | 44.99 | 12.543 | 12.0 | 0.179 | 86.55 | 34 | 64.45 | 17.29 | 7.9 | 0.343 | 123.6 | 530.1 | 24236 | 18706 |  | 167.3 | 17.2 |  | 680 | 13.3 |  | 30.1 | 59 | 10.9 |
| **PO2** | 44.993 | 12.556 | 20.5 | 0.269 | 75.13 | 39.66 | 61.47 | 21.81 | 8.39 | 0.359 | 116.2 | 577.6 | 24194 | 15818 |  | 123.5 | 11.3 |  | 680.6 | 16.7 |  | 14.5 | 68.6 | 16.9 |
| **PO3** | 44.968 | 12.571 | 9.5 | 0.1 | 105.35 | 47.42 | 89.2 | 21.15 | 7.36 | 0.394 | 136.6 | 654.2 | 25747 | 33392 |  | 148.4 | 23.4 |  | 847.3 | 13.1 |  | 24.2 | 63.2 | 12.7 |
| **PO4** | 44.971 | 12.576 | 15.0 | 0.1 | 112.06 | 53.86 | 96.25 | 24.24 | 8.31 | 0.468 | 149.9 | 764.6 | 27752 | 20164 |  | 145.7 | 25.1 |  | 834.9 | 13.5 |  | 15.9 | 67.5 | 16.6 |
| **PO5** | 44.974 | 12.581 | 19.0 | 0.117 | 107.61 | 52.07 | 91.87 | 23.84 | 8.68 | 0.463 | 137.7 | 673.4 | 26428 | 19381 |  | 152.1 | 26.1 |  | 770.6 | 13.9 |  | 14.3 | 68.1 | 17.6 |
| **PO6** | 44.955 | 12.573 | 10.5 | 0.107 | 103.32 | 51.37 | 82.04 | 22.43 | 8.01 | 0.343 | 133.9 | 708.2 | 30641 | 28719 |  | 183.4 | 28.1 |  | 768.9 | 14.4 |  | 11.3 | 72.9 | 15.8 |
| **PO7** | 44.952 | 12.585 | 14.0 | 0.136 | 99.53 | 51.84 | 86.95 | 22.65 | 8.43 | 0.503 | 143.4 | 692 | 27845 | 19352 |  | 170.9 | 22.3 |  | 795.2 | 14.6 |  | 12.7 | 72.1 | 15.2 |
| **PO8** | 44.929 | 12.558 | 11.0 | 0.201 | 80.09 | 34.67 | 67.84 | 17.1 | 6.13 | 0.347 | 107.5 | 614.9 | 24294 | 17509 |  | 89.2 | 14.6 |  | 737.4 | 12.4 |  | 26.1 | 59.6 | 14.3 |
| **PO9** | 44.919 | 12.573 | 14.6 | 0.139 | 102.23 | 50.26 | 81.63 | 24.04 | 8.82 | 0.343 | 140.8 | 740.7 | 32112 | 28620 |  | 143.7 | 17.2 |  | 724.7 | 12.8 |  | 7.1 | 73.7 | 19.3 |
| **PO10** | 44.891 | 12.538 | 14.8 | 0.176 | 70.15 | 23.88 | 55.73 | 12.92 | 5.88 | 0.231 | 91.1 | 554.9 | 21148 | 14629 |  | 194.2 | 19.1 |  | 679.6 | 7.3 |  | 22.7 | 64.1 | 13.3 |
| **PO11** | 44.886 | 12.558 | 17.5 | 0.285 | 86.51 | 41.85 | 66.09 | 23.48 | 8.68 | 0.383 | 125 | 678.8 | 26514 | 21959 |  | 183.8 | 13 |  | 682.6 | 12 |  | 11.7 | 72 | 16.3 |
| **PO12** | 44.867 | 12.519 | 13.5 | 0.164 | 90.53 | 40.83 | 73.14 | 20.47 | 7.46 | 0.355 | 120.7 | 661.5 | 27692 | 23172 |  | 119.2 | 15.1 |  | 774 | 13.4 |  | 8.6 | 71 | 20.5 |
| **PO13** | 44.861 | 12.544 | 18.8 | 0.291 | 92.9 | 49.7 | 75.3 | 27.11 | 9.85 | 0.348 | 136.7 | 726.3 | 29227 | 23087 |  | 233.8 | 21.7 |  | 652.2 | 16.2 |  | 3.9 | 69.5 | 26.6 |
| **PO14** | 44.832 | 12.487 | 10.5 | 0.083 | 92.6 | 37.11 | 74.71 | 18.13 | 7.01 | 0.242 | 106.4 | 681.8 | 26993 | 21203 |  | 141.1 | 19.7 |  | 741 | 13 |  | 10.7 | 68.1 | 21.2 |
| **PO15** | 44.821 | 12.513 | 18.0 | 0.209 | 93.53 | 41.18 | 73.37 | 23.2 | 9.23 | 0.243 | 122.7 | 720.3 | 29031 | 24063 |  | 169.2 | 12 |  | 614.6 | 15.4 |  | 9.7 | 68.7 | 21.6 |
| **PO16** | 44.796 | 12.457 | 10.5 | 0.135 | 108.97 | 56.27 | 91.77 | 25.8 | 8.35 | 0.369 | 146.2 | 744.5 | 30442 | 24386 |  | 118.4 | 18 |  | 742 | 14.9 |  | 9.9 | 70.5 | 19.6 |
| **PO17** | 44.786 | 12.485 | 18.5 | 0.125 | 102.9 | 54.36 | 88.04 | 24.51 | 8.13 | 0.348 | 138.2 | 715.4 | 29512 | 22787 |  | 140.1 | 24.5 |  | 727.1 | 14.4 |  | 8.9 | 70.7 | 20.4 |
| **PO18** | 44.78 | 12.437 | 11.5 | 0.056 | 88.34 | 30.72 | 73.93 | 14.79 | 5.1 | 0.255 | 96.9 | 641.2 | 24767 | 19605 |  | 83 | 12 |  | 596.6 | 12.5 |  | 9.1 | 72.3 | 18.6 |
| **PO19** | 44.764 | 12.461 | 18.5 | 0.199 | 79.16 | 32.51 | 66.5 | 18.11 | 7.2 | 0.248 | 110.4 | 627.8 | 25471 | 18536 |  | 104.2 | 15.2 |  | 627.8 | 14.7 |  | 7.8 | 72.5 | 19.7 |
|  |  |  |  |  |  |  |  |  |  |  |  |  |  |  |  |  |  |  |  |  |  |  |  |  |

**Supplementary Table 2.** Position (as latitude and longitude) of the sampling stations within the Mar Piccolo of Taranto and concentration of heavy metals (as mg Kg^-1^), organic matter content (expressed as % of LOI, loss on ignition), PAHs and PCBs (as ng gr^-1^) and grain size (expressed as % of sands, silt and clay) in the sediments.

|  | | **N** | | **E** | | **mt** | | **mg Kg^-1^** | | | | | | | | | | | | | | | | | | | | | |  | | **ng gr^-1^** | | | |  | | **mg Kg^-1^** | | **%** | |  | | **%** | | | | | |
| --- | --- | --- | --- | --- | --- | --- | --- | --- | --- | --- | --- | --- | --- | --- | --- | --- | --- | --- | --- | --- | --- | --- | --- | --- | --- | --- | --- | --- | --- | --- | --- | --- | --- | --- | --- | --- | --- | --- | --- | --- | --- | --- | --- | --- | --- | --- | --- | --- | --- |
| **Station** | | **Lat** | | **Long** | | **Depth** | | **Hg** | | **Cr** | | **Cu** | | **Ni** | | **Pb** | | **As** | | **Cd** | | **Zn** | | **Mn** | | **Fe** | | **Al** | |  | | **PAHs** | | **PCBs** | |  | | **P** | | **LOI** | |  | | **Sand** | | **Silt** | | **Clay** | |
| **TA1** | 40.4781 | | 17.2369 | | 11.6 | | 0.98 | | 15.43 | | 17.53 | | 8.27 | | 36.9 | | 8.15 | | 0.247 | | 56.8 | | 190.1 | | 6768 | | 4906 | |  | | 1645.75 | | 277.34 | |  | | 650.4 | | 8.46 | |  | | 78.8 | | 16.6 | | 4.6 | |  |
| **TA4** | 40.4789 | | 17.2453 | | 11.7 | | 6.54 | | 57.18 | | 86.92 | | 41.96 | | 113.43 | | 16.71 | | 0.346 | | 260.5 | | 401.2 | | 29510 | | 22501 | |  | | 2140.52 | | 503.72 | |  | | 757.3 | | 20.91 | |  | | 23.2 | | 61 | | 15.8 | |  |
| **TA5** | 40.4794 | | 17.2606 | | 11.4 | | 8.98 | | 72.91 | | 119.02 | | 50.4 | | 129.79 | | 22.14 | | 0.489 | | 353.5 | | 420.8 | | 34141 | | 27719 | |  | | 1623.96 | | 1045.06 | |  | | 704.6 | | 20.58 | |  | | 19.9 | | 63.4 | | 16.7 | |  |
| **TA7** | 40.4839 | | 17.2461 | | 11.3 | | 3.98 | | 52.19 | | 237.72 | | 39.41 | | 104.39 | | 16.04 | | 0.349 | | 312.7 | | 379 | | 27759 | | 19483 | |  | | 1756.14 | | 503.14 | |  | | 699.8 | | 20.41 | |  | | 19.4 | | 61.1 | | 19.5 | |  |
| **TA8** | 40.4889 | | 17.2558 | | 11.2 | | 5.26 | | 70.98 | | 80.7 | | 46.85 | | 96.16 | | 14.64 | | 0.351 | | 258.9 | | 391.2 | | 32867 | | 28521 | |  | | 2280.38 | | 622.52 | |  | | 754.3 | | 22.37 | |  | | 20 | | 62 | | 18.1 | |  |
| **TA9** | 40.4969 | | 17.2619 | | 7.3 | | 4.39 | | 51.46 | | 118.95 | | 37.96 | | 125.58 | | 13.62 | | 0.723 | | 312.1 | | 348.3 | | 25056 | | 19958 | |  | | 1762.03 | | 710.04 | |  | | 603.8 | | 22.09 | |  | | 27.9 | | 59.5 | | 12.6 | |  |
| **TA10** | 40.48 | | 17.2783 | | 8.2 | | 1.89 | | 64.47 | | 66.48 | | 47.96 | | 53.99 | | 11.27 | | 0.558 | | 254.3 | | 300.1 | | 30597 | | 32359 | |  | | 356.95 | | 137.61 | |  | | 561.1 | | 21.22 | |  | | 27.6 | | 57.9 | | 14.5 | |  |
| **TA11** | 40.485 | | 17.2803 | | 6.3 | | 0.92 | | 55.55 | | 56.57 | | 45.33 | | 40.9 | | 11.36 | | 0.568 | | 229.5 | | 293.1 | | 28924 | | 26822 | |  | | 527.73 | | 98.02 | |  | | 577.1 | | 21.64 | |  | | 29.4 | | 58.2 | | 12.4 | |  |
| **TA12** | 40.4806 | | 17.2953 | | 8.0 | | 0.73 | | 62.77 | | 52.15 | | 45.47 | | 33.41 | | 8.35 | | 0.358 | | 167.1 | | 262.5 | | 28115 | | 33544 | |  | | 156.53 | | 82.94 | |  | | 575.2 | | 20.92 | |  | | 25.9 | | 54.2 | | 19.9 | |  |
| **TA13** | 40.4831 | | 17.2583 | | 11.4 | | 6.69 | | 71.56 | | 105.5 | | 49.67 | | 124.42 | | 19.87 | | 0.473 | | 291 | | 460.1 | | 31153 | | 27771 | |  | | 4690.13 | | 625.87 | |  | | 779.4 | | 22.2 | |  | | 19.8 | | 63.2 | | 17 | |  |
| **TA14** | 40.4828 | | 17.2403 | | 11.0 | | 3.03 | | 49.78 | | 75.8 | | 32.83 | | 69.11 | | 11.82 | | 0.239 | | 372.4 | | 407 | | 33435 | | 27681 | |  | | 3043.23 | | 527.68 | |  | | 685.2 | | 20.01 | |  | | 30.4 | | 53.1 | | 16.5 | |  |

**Supplementary Table 3.** Concentration of PCBs and PAHs congeners (as ng gr^-1^) in the sediments of the Po River Prodelta site.

|  | **PO1** | **PO2** | **PO3** | **PO4** | **PO5** | **PO6** | **PO7** | **PO8** | **PO9** | **PO10** | **PO11** | **PO12** | **PO13** | **PO14** | **PO15** | **PO16** | **PO17** | **PO18** | **PO19** |
| --- | --- | --- | --- | --- | --- | --- | --- | --- | --- | --- | --- | --- | --- | --- | --- | --- | --- | --- | --- |
| *PCBs congeners* |  |  |  |  |  |  |  |  |  |  |  |  |  |  |  |  |  |  |  |
| **18** | 0.00 | 0.00 | 0.00 | 0.00 | 0.00 | 0.74 | 0.63 | 0.67 | 0.70 | 0.00 | 0.00 | 0.00 | 0.00 | 0.00 | 0.00 | 0.00 | 0.00 | 0.00 | 0.00 |
| **28+31** | 0.39 | 0.47 | 0.40 | 0.23 | 0.60 | 0.00 | 0.00 | 0.44 | 0.22 | 0.00 | 0.00 | 0.00 | 0.00 | 0.60 | 0.00 | 0.00 | 0.00 | 0.00 | 0.00 |
| **52** | 0.00 | 0.00 | 0.70 | 0.81 | 0.59 | 0.63 | 1.24 | 0.00 | 0.00 | 0.00 | 0.00 | 0.00 | 0.00 | 1.23 | 0.00 | 0.00 | 0.00 | 0.00 | 0.00 |
| **44** | 0.00 | 0.00 | 0.00 | 0.52 | 0.00 | 0.00 | 0.00 | 0.00 | 0.00 | 0.00 | 0.00 | 0.00 | 0.00 | 0.00 | 0.00 | 0.00 | 0.00 | 0.00 | 0.00 |
| **95** | 0.00 | 0.55 | 1.32 | 1.05 | 1.01 | 0.91 | 0.56 | 0.87 | 0.26 | 0.44 | 0.00 | 0.27 | 0.24 | 0.86 | 0.00 | 0.92 | 0.51 | 0.23 | 0.75 |
| **101** | 0.75 | 0.57 | 1.35 | 1.86 | 1.93 | 1.59 | 1.11 | 0.73 | 0.85 | 1.05 | 0.77 | 0.00 | 0.86 | 1.73 | 0.91 | 1.20 | 1.64 | 0.53 | 1.44 |
| **99** | 0.49 | 0.00 | 0.48 | 0.30 | 0.56 | 0.46 | 0.44 | 0.15 | 0.46 | 0.37 | 0.20 | 0.66 | 1.14 | 0.31 | 0.39 | 0.42 | 0.44 | 0.30 | 0.25 |
| **81** | 0.00 | 0.00 | 0.69 | 0.59 | 0.73 | 1.04 | 0.66 | 0.33 | 1.23 | 0.00 | 1.36 | 0.63 | 0.57 | 0.00 | 0.00 | 0.76 | 0.80 | 0.00 | 0.83 |
| **110** | 0.58 | 0.22 | 0.78 | 0.65 | 1.12 | 0.52 | 0.60 | 0.40 | 0.39 | 1.39 | 0.40 | 0.25 | 0.74 | 0.58 | 0.24 | 0.63 | 0.89 | 0.36 | 1.01 |
| **77** | 0.00 | 0.00 | 0.00 | 0.00 | 0.00 | 0.00 | 0.00 | 0.00 | 0.00 | 0.00 | 0.00 | 0.00 | 0.00 | 0.00 | 0.00 | 0.00 | 0.00 | 0.00 | 0.00 |
| **151** | 0.40 | 0.31 | 0.61 | 0.59 | 0.53 | 0.57 | 0.46 | 0.34 | 0.38 | 0.38 | 0.25 | 1.10 | 0.47 | 0.35 | 0.29 | 0.36 | 0.57 | 0.26 | 0.57 |
| **123+149** | 1.30 | 1.06 | 2.10 | 2.12 | 2.15 | 2.32 | 2.04 | 1.23 | 1.36 | 1.95 | 0.99 | 1.14 | 1.77 | 1.55 | 1.03 | 1.47 | 2.27 | 1.23 | 1.55 |
| **118** | 0.65 | 0.20 | 0.35 | 0.31 | 0.30 | 0.83 | 1.34 | 0.88 | 0.49 | 0.41 | 0.27 | 0.49 | 0.56 | 0.41 | 0.00 | 0.00 | 0.37 | 0.44 | 0.72 |
| **114** | 0.00 | 0.00 | 0.00 | 0.00 | 0.17 | 0.32 | 0.14 | 0.14 | 0.00 | 0.00 | 0.00 | 0.00 | 0.00 | 0.00 | 0.00 | 0.00 | 0.00 | 0.00 | 0.18 |
| **146** | 0.00 | 0.23 | 0.46 | 0.52 | 0.44 | 0.45 | 0.50 | 0.00 | 0.36 | 0.36 | 0.28 | 0.28 | 0.46 | 0.35 | 0.28 | 0.26 | 0.38 | 0.23 | 0.39 |
| **153** | 3.35 | 2.47 | 4.39 | 4.29 | 4.07 | 4.87 | 3.67 | 2.63 | 2.99 | 3.34 | 2.53 | 3.00 | 4.12 | 4.10 | 2.57 | 3.49 | 4.39 | 2.39 | 0.47 |
| **105** | 0.00 | 0.11 | 0.00 | 0.00 | 0.00 | 0.00 | 0.00 | 0.00 | 0.00 | 0.22 | 0.00 | 0.00 | 0.25 | 0.00 | 0.00 | 0.00 | 0.00 | 0.00 | 0.00 |
| **138** | 2.62 | 1.68 | 1.60 | 3.84 | 3.95 | 4.31 | 3.27 | 2.27 | 2.55 | 3.20 | 1.84 | 2.83 | 3.16 | 2.83 | 2.09 | 2.75 | 3.99 | 2.35 | 2.65 |
| **126** | 0.00 | 0.00 | 0.00 | 0.00 | 0.00 | 0.00 | 0.00 | 0.00 | 0.00 | 0.00 | 0.00 | 0.00 | 0.00 | 0.00 | 0.00 | 0.00 | 0.00 | 0.00 | 0.00 |
| **183** | 1.27 | 0.87 | 1.54 | 1.68 | 1.58 | 1.67 | 1.32 | 0.90 | 1.31 | 1.23 | 1.06 | 1.10 | 1.50 | 1.05 | 1.12 | 1.30 | 1.68 | 0.82 | 0.83 |
| **187** | 0.54 | 0.33 | 0.66 | 0.82 | 0.75 | 0.81 | 0.63 | 0.46 | 0.58 | 0.54 | 0.45 | 0.54 | 0.57 | 0.42 | 0.39 | 0.56 | 0.75 | 0.32 | 0.25 |
| **128** | 0.00 | 0.00 | 0.00 | 0.00 | 0.00 | 0.00 | 0.00 | 0.00 | 0.00 | 0.12 | 0.00 | 0.00 | 0.19 | 0.00 | 0.00 | 0.00 | 0.21 | 0.00 | 0.00 |
| **167** | 0.20 | 0.00 | 0.76 | 0.13 | 0.11 | 0.96 | 0.48 | 0.00 | 0.10 | 0.00 | 0.42 | 0.33 | 0.27 | 0.00 | 0.00 | 0.21 | 0.30 | 0.24 | 0.00 |
| **177** | 0.70 | 0.40 | 0.68 | 0.59 | 0.81 | 0.69 | 0.00 | 0.39 | 0.23 | 0.37 | 0.00 | 0.00 | 0.61 | 0.54 | 0.49 | 0.57 | 0.73 | 0.00 | 0.40 |
| **156** | 0.00 | 0.00 | 0.18 | 0.00 | 0.00 | 0.00 | 0.00 | 0.00 | 0.00 | 0.00 | 0.14 | 0.00 | 0.00 | 0.00 | 0.00 | 0.00 | 0.12 | 0.00 | 0.00 |
| **157** | 0.48 | 0.10 | 0.23 | 0.20 | 0.36 | 0.21 | 0.15 | 0.00 | 0.14 | 0.00 | 0.00 | 0.00 | 0.23 | 0.18 | 0.00 | 0.14 | 0.37 | 0.00 | 0.22 |
| **180** | 2.60 | 1.33 | 2.90 | 3.05 | 3.40 | 3.04 | 2.32 | 1.31 | 2.00 | 2.73 | 1.38 | 1.57 | 2.87 | 1.99 | 1.69 | 2.15 | 3.32 | 1.75 | 1.41 |
| **169** | 0.00 | 0.00 | 0.00 | 0.00 | 0.00 | 0.00 | 0.00 | 0.00 | 0.00 | 0.00 | 0.00 | 0.00 | 0.00 | 0.00 | 0.00 | 0.00 | 0.00 | 0.00 | 0.00 |
| **170** | 0.86 | 0.42 | 0.96 | 0.96 | 0.95 | 1.19 | 0.73 | 0.42 | 0.63 | 0.91 | 0.61 | 0.84 | 0.95 | 0.61 | 0.52 | 0.81 | 0.83 | 0.61 | 1.31 |
| **189** | 0.00 | 0.00 | 0.28 | 0.00 | 0.00 | 0.00 | 0.00 | 0.00 | 0.00 | 0.13 | 0.10 | 0.10 | 0.16 | 0.00 | 0.00 | 0.00 | 0.00 | 0.00 | 0.00 |
| *PAHs congeners* |  |  |  |  |  |  |  |  |  |  |  |  |  |  |  |  |  |  |  |
| **Naphthalene** | 0.00 | 0.00 | 0.00 | 0.00 | 0.00 | 0.00 | 0.00 | 0.00 | 0.00 | 0.00 | 0.00 | 0.00 | 0.00 | 0.00 | 0.00 | 0.00 | 0.00 | 0.00 | 0.00 |
| **Acenaphthylene** | 0.00 | 0.00 | 0.00 | 0.00 | 0.00 | 0.00 | 0.00 | 0.00 | 0.00 | 0.00 | 0.00 | 0.00 | 0.00 | 0.00 | 0.00 | 0.00 | 0.00 | 0.00 | 0.00 |
| **Acenaphthene** | 0.84 | 0.45 | 0.38 | 0.17 | 0.55 | 0.45 | 0.59 | 0.38 | 0.69 | 0.64 | 0.46 | 0.00 | 1.29 | 1.00 | 1.16 | 0.61 | 1.09 | 0.37 | 0.26 |
| **Fluorene** | 1.83 | 1.23 | 1.18 | 1.04 | 1.48 | 1.50 | 1.88 | 1.27 | 2.29 | 1.03 | 2.34 | 2.08 | 2.78 | 2.36 | 3.20 | 1.59 | 2.46 | 1.53 | 1.78 |
| **Phenanthrene** | 17.04 | 13.56 | 12.54 | 12.68 | 14.74 | 16.20 | 17.57 | 11.52 | 15.55 | 12.69 | 16.67 | 13.36 | 19.59 | 18.16 | 18.32 | 12.67 | 14.74 | 10.04 | 12.90 |
| **Anthracene** | 3.51 | 2.20 | 2.60 | 2.57 | 2.55 | 3.41 | 2.74 | 1.60 | 2.43 | 4.45 | 3.15 | 2.26 | 4.49 | 2.36 | 3.25 | 2.28 | 2.60 | 1.69 | 2.02 |
| **Fluoranthene** | 31.72 | 22.86 | 24.19 | 25.70 | 26.73 | 31.18 | 28.77 | 14.92 | 21.45 | 33.83 | 32.64 | 18.18 | 39.89 | 23.02 | 29.48 | 20.45 | 26.05 | 16.88 | 21.01 |
| **Pyrene** | 26.15 | 17.85 | 20.75 | 21.61 | 24.41 | 26.22 | 22.32 | 12.63 | 17.29 | 36.26 | 29.77 | 15.00 | 35.35 | 14.45 | 21.23 | 14.78 | 17.45 | 9.53 | 12.08 |
| **Benzo[a]anthracene** | 16.62 | 9.53 | 12.68 | 12.72 | 15.06 | 18.76 | 16.44 | 5.49 | 10.55 | 17.94 | 19.77 | 9.44 | 22.18 | 10.57 | 14.24 | 9.76 | 11.45 | 6.55 | 8.31 |
| **Chrysene** | 20.21 | 14.46 | 18.12 | 18.51 | 18.90 | 24.11 | 21.28 | 9.87 | 16.81 | 24.01 | 23.19 | 15.71 | 28.03 | 17.46 | 22.16 | 15.05 | 17.94 | 11.00 | 12.43 |
| **Benzo[b]fluoranthene** | 17.28 | 13.83 | 16.51 | 17.17 | 17.03 | 20.50 | 19.47 | 10.11 | 17.03 | 10.35 | 21.01 | 15.10 | 25.69 | 13.39 | 20.60 | 12.65 | 14.87 | 10.23 | 13.91 |
| **Benzo[k]fluoranthene** | 7.83 | 5.82 | 7.17 | 7.47 | 7.33 | 8.36 | 7.85 | 3.41 | 6.25 | 10.40 | 8.10 | 5.10 | 10.50 | 5.04 | 7.47 | 4.96 | 5.52 | 3.78 | 4.53 |
| **Benzo[a]pyrene** | 10.16 | 5.03 | 9.02 | 8.64 | 7.91 | 11.70 | 8.20 | 5.40 | 10.08 | 20.08 | 10.96 | 7.83 | 16.63 | 9.23 | 10.56 | 6.26 | 9.12 | 3.10 | 6.37 |
| **Dibenz[a,h]anthracene** | 1.90 | 0.48 | 0.60 | 0.39 | 0.23 | 0.25 | 0.19 | 0.16 | 0.44 | 0.48 | 0.23 | 0.25 | 0.72 | 0.23 | 0.23 | 0.00 | 0.19 | 0.26 | 0.20 |
| **Benzo[g,h,j]perylene + Indeno[1,2,3-cd]pyrene** | 12.24 | 16.19 | 22.64 | 17.04 | 15.18 | 20.73 | 23.61 | 12.45 | 22.84 | 22.05 | 15.46 | 14.91 | 26.62 | 23.85 | 17.26 | 17.37 | 16.64 | 8.08 | 8.41 |

**Supplementary Table 4.** Concentration of PCBs and PAHs congeners (as ng gr^-1^) in the sediments of the Mar Piccolo of Taranto site.

|  | **TA01** | **TA04** | **TA05** | **TA07** | **TA08** | **TA09** | **TA10** | **TA11** | **TA12** | **TA13** | **TA14** |
| --- | --- | --- | --- | --- | --- | --- | --- | --- | --- | --- | --- |
| *PCBs congeners* |  |  |  |  |  |  |  |  |  |  |  |
| **18** | 0.00 | 0.38 | 0.77 | 0.83 | 0.00 | 2.96 | 1.07 | 0.00 | 0.00 | 0.00 | 2.69 |
| **28+31** | 0.00 | 3.23 | 4.09 | 4.84 | 3.84 | 1.75 | 0.56 | 0.33 | 0.53 | 2.69 | 2.20 |
| **52** | 4.95 | 14.19 | 16.99 | 9.81 | 9.06 | 21.23 | 2.94 | 1.19 | 1.12 | 12.25 | 12.63 |
| **44** | 4.84 | 17.35 | 21.63 | 8.87 | 7.82 | 7.27 | 0.22 | 0.48 | 0.48 | 9.35 | 2.68 |
| **95** | 14.15 | 21.35 | 54.26 | 22.22 | 28.84 | 52.12 | 2.60 | 4.75 | 3.37 | 51.03 | 28.84 |
| **101** | 21.06 | 26.24 | 77.87 | 32.33 | 50.65 | 73.62 | 8.73 | 7.84 | 5.46 | 42.05 | 38.38 |
| **99** | 10.89 | 20.48 | 47.65 | 22.75 | 31.25 | 40.95 | 7.80 | 6.28 | 5.58 | 29.56 | 30.45 |
| **81** | 0.00 | 0.00 | 0.00 | 0.00 | 0.00 | 0.00 | 0.00 | 0.00 | 0.00 | 0.00 | 0.00 |
| **110** | 8.76 | 12.91 | 38.17 | 12.35 | 25.59 | 38.06 | 3.06 | 2.76 | 2.56 | 19.61 | 16.99 |
| **77** | 0.00 | 0.00 | 0.00 | 0.00 | 0.00 | 0.00 | 0.00 | 0.00 | 0.00 | 0.00 | 0.00 |
| **151** | 5.61 | 12.04 | 26.87 | 13.07 | 15.77 | 14.56 | 3.04 | 2.03 | 1.85 | 15.59 | 13.90 |
| **123+149** | 18.79 | 33.54 | 78.40 | 41.25 | 47.12 | 55.80 | 11.27 | 7.86 | 7.60 | 46.01 | 41.46 |
| **118** | 10.43 | 19.30 | 45.08 | 18.61 | 30.80 | 30.93 | 8.99 | 3.72 | 3.52 | 25.33 | 24.26 |
| **114** | 3.91 | 7.11 | 16.98 | 4.39 | 6.57 | 14.08 | 0.17 | 0.99 | 0.65 | 8.48 | 5.31 |
| **146** | 5.66 | 13.66 | 30.94 | 12.94 | 18.75 | 18.89 | 5.03 | 3.93 | 3.03 | 20.16 | 14.53 |
| **153** | 35.70 | 55.45 | 126.65 | 67.35 | 80.32 | 81.85 | 23.80 | 18.37 | 13.82 | 72.98 | 65.23 |
| **105** | 1.77 | 6.69 | 15.77 | 7.46 | 10.26 | 17.53 | 2.24 | 0.53 | 1.41 | 11.66 | 9.71 |
| **138** | 28.61 | 47.02 | 103.31 | 51.74 | 66.04 | 78.31 | 12.95 | 10.80 | 8.53 | 58.68 | 55.73 |
| **126** | 2.89 | 6.42 | 14.07 | 3.81 | 7.29 | 4.62 | 5.07 | 0.00 | 0.00 | 0.00 | 0.00 |
| **183** | 17.02 | 39.35 | 71.82 | 41.69 | 42.79 | 31.57 | 12.82 | 9.87 | 8.77 | 45.05 | 42.08 |
| **187** | 8.50 | 14.60 | 27.70 | 15.87 | 16.69 | 11.84 | 2.57 | 2.22 | 1.65 | 17.29 | 13.36 |
| **128** | 3.21 | 5.92 | 17.46 | 3.16 | 10.06 | 11.61 | 0.62 | 0.44 | 0.38 | 10.14 | 7.04 |
| **167** | 4.90 | 10.25 | 17.64 | 6.80 | 10.14 | 5.98 | 0.77 | 0.30 | 0.45 | 10.13 | 5.72 |
| **177** | 9.58 | 19.89 | 20.45 | 10.36 | 13.24 | 14.25 | 3.91 | 1.01 | 1.25 | 16.49 | 11.15 |
| **156** | 6.78 | 15.97 | 30.42 | 7.80 | 14.94 | 14.31 | 1.57 | 1.92 | 1.13 | 16.38 | 8.13 |
| **157** | 4.86 | 9.23 | 16.96 | 7.56 | 11.10 | 6.98 | 1.24 | 0.79 | 0.24 | 1.06 | 5.94 |
| **180** | 32.04 | 53.48 | 92.57 | 56.99 | 42.09 | 39.84 | 11.67 | 7.52 | 7.54 | 58.83 | 49.44 |
| **169** | 0.00 | 0.00 | 0.00 | 0.00 | 0.00 | 2.64 | 0.00 | 0.00 | 0.00 | 1.35 | 0.00 |
| **170** | 10.84 | 16.65 | 27.26 | 16.90 | 20.40 | 16.11 | 2.89 | 2.08 | 2.03 | 21.25 | 16.23 |
| **189** | 1.56 | 1.04 | 3.27 | 1.39 | 1.09 | 0.37 | 0.00 | 0.00 | 0.00 | 2.46 | 3.60 |
| *PAHs congeners* |  |  |  |  |  |  |  |  |  |  |  |
| **Naphthalene** | 0.00 | 0.00 | 0.00 | 0.00 | 0.00 | 0.00 | 0.00 | 0.00 | 0.00 | 0.00 | 0.00 |
| **Acenaphthylene** | 0.00 | 0.00 | 0.00 | 0.00 | 0.00 | 0.00 | 0.00 | 0.00 | 0.00 | 0.00 | 0.00 |
| **Acenaphthene** | 1.34 | 21.04 | 5.38 | 8.50 | 5.78 | 2.85 | 3.64 | 3.20 | 0.22 | 7.19 | 4.45 |
| **Fluorene** | 13.93 | 18.00 | 13.63 | 8.74 | 15.95 | 7.54 | 3.03 | 3.36 | 1.06 | 15.04 | 16.79 |
| **Phenanthrene** | 109.58 | 134.39 | 98.74 | 73.75 | 129.67 | 63.71 | 16.02 | 28.08 | 8.33 | 197.36 | 168.23 |
| **Anthracene** | 52.94 | 59.04 | 38.88 | 31.33 | 52.35 | 22.16 | 6.95 | 10.90 | 2.88 | 89.39 | 69.90 |
| **Fluoranthene** | 251.05 | 332.83 | 204.58 | 234.91 | 327.47 | 226.26 | 48.03 | 85.72 | 24.51 | 841.04 | 497.15 |
| **Pyrene** | 268.23 | 371.91 | 232.41 | 262.21 | 356.71 | 253.88 | 55.19 | 95.67 | 24.23 | 940.25 | 555.19 |
| **Benzo[a]anthracene** | 131.07 | 145.38 | 87.57 | 122.94 | 158.64 | 107.47 | 22.35 | 41.59 | 9.96 | 343.81 | 233.12 |
| **Chrysene** | 151.35 | 173.78 | 92.44 | 143.04 | 184.81 | 144.25 | 22.42 | 47.19 | 10.61 | 392.70 | 271.11 |
| **Benzo[b]fluoranthene** | 133.36 | 242.07 | 217.64 | 220.08 | 260.60 | 213.63 | 48.51 | 55.82 | 18.82 | 505.72 | 304.92 |
| **Benzo[k]fluoranthene** | 67.18 | 101.22 | 97.36 | 98.73 | 126.03 | 102.19 | 18.63 | 23.91 | 8.75 | 174.53 | 146.33 |
| **Benzo[a]pyrene** | 140.80 | 91.28 | 91.74 | 111.14 | 104.55 | 159.17 | 32.73 | 42.79 | 11.16 | 258.22 | 171.73 |
| **Dibenz[a,h]anthracene** | 4.24 | 8.60 | 8.65 | 17.31 | 17.88 | 0.94 | 4.74 | 0.74 | 0.00 | 3.33 | 1.24 |
| **Benzo[g,h,j]perylene + Indeno[1,2,3-cd]pyrene** | 320.69 | 440.97 | 434.93 | 423.47 | 539.93 | 457.99 | 74.71 | 88.77 | 36.01 | 921.56 | 603.08 |

**Supplementary Table 5.** Output of the DistLM analysis performed at the Phylum level for all sites. Reported are the results of the Marginal Tests. SS: Sum of square. Prop: percentage of variance explained by each variable. P values having statistical significance are written in bold and italic.

| **MARGINAL TESTS** |  |  |  |  |
| --- | --- | --- | --- | --- |
|  |  |  |  |  |
| **Variable** | **SS (trace)** | **Pseudo-F** | **P** | **Prop.** |
| Al | 244.89 | 1.0944 | 0.3454 | 0.038 |
| As | 1572.8 | 8.9194 | ***0.0002*** | 0.242 |
| Cd | 336.1 | 1.5243 | 0.2018 | 0.052 |
| Cr | 1934 | 11.833 | ***0.0002*** | 0.297 |
| Cu | 884.16 | 4.4004 | ***0.003*** | 0.136 |
| Fe | 78.684 | 0.34256 | 0.8462 | 0.012 |
| Hg | 1672.7 | 9.6821 | ***0.0002*** | 0.257 |
| LOI% | 1613.6 | 9.2272 | ***0.0002*** | 0.248 |
| Mn | 2221.9 | 14.508 | ***0.0002*** | 0.341 |
| Ni | 2114 | 13.464 | ***0.0002*** | 0.325 |
| P | 734.85 | 3.5627 | ***0.0168*** | 0.113 |
| PAHs | 1575.2 | 8.9375 | ***0.0002*** | 0.242 |
| Pb | 1822.7 | 10.888 | ***0.0002*** | 0.280 |
| PCBs | 1785.7 | 10.583 | ***0.0002*** | 0.274 |
| silt | 939.71 | 4.7235 | ***0.0014*** | 0.144 |
| Zn | 1649.9 | 9.5052 | ***0.0002*** | 0.253 |
| res.df: 28 |  |  |  |  |
| Total SS(trace): 6510.2 |  |  |  |  |

**Supplementary Table 6.** Output of the DistLM analysis at the OTU level for all sites. Reported are the results of the Marginal Tests. SS: Sum of square. Prop: percentage of variance explained by each variable. P values having statistical significance are written in bold and italic.

| **MARGINAL TESTS** |  |  |  |  |
| --- | --- | --- | --- | --- |
|  |  |  |  |  |
| **Variable** | **SS(trace)** | **Pseudo-F** | **P** | **Prop.** |
| Al | 3213.4 | 1.2444 | 0.218 | 0.043 |
| As | 15308 | 7.1187 | ***0.001*** | 0.203 |
| Cd | 4034 | 1.5801 | 0.121 | 0.053 |
| Cr | 18653 | 9.185 | ***0.001*** | 0.247 |
| Cu | 8948.2 | 3.7638 | ***0.001*** | 0.118 |
| Fe | 2061 | 0.7856 | 0.605 | 0.027 |
| Hg | 15852 | 7.4392 | ***0.001*** | 0.210 |
| LOI% | 15192 | 7.0515 | ***0.001*** | 0.201 |
| Mn | 20207 | 10.23 | ***0.001*** | 0.268 |
| Ni | 20816 | 10.655 | ***0.001*** | 0.276 |
| P | 7735.8 | 3.1956 | ***0.007*** | 0.102 |
| PAHs | 14999 | 6.9398 | ***0.001*** | 0.199 |
| Pb | 17098 | 8.1949 | ***0.001*** | 0.226 |
| PCBs | 16287 | 7.6994 | ***0.001*** | 0.216 |
| silt | 8745.8 | 3.6675 | ***0.001*** | 0.116 |
| Zn | 15623 | 7.3034 | ***0.001*** | 0.207 |
| res.df: 28 |  |  |  |  |
| Total SS(trace): 75517 |  |  |  |  |

**Supplementary Table 7.** Output of dbRDA correlations. Reported are the coordinates scores for each station in the Po River Prodelta (A) and the correlations between each environmental variable, chemical contaminant and dbRDA axis (B).

| **A dbRDA coordinate scores** | | | | | | |  | | |  | | |  | | |  | | |  | | |  | | |  | | |  | | | |  | | |  | | |  | | |  | | |  | | | |  |
| --- | --- | --- | --- | --- | --- | --- | --- | --- | --- | --- | --- | --- | --- | --- | --- | --- | --- | --- | --- | --- | --- | --- | --- | --- | --- | --- | --- | --- | --- | --- | --- | --- | --- | --- | --- | --- | --- | --- | --- | --- | --- | --- | --- | --- | --- | --- | --- | --- |
| **Sample** | **dbRDA1** | **dbRDA2** | | | **dbRDA3** | | | **dbRDA4** | | | **dbRDA5** | | | **dbRDA6** | | | | **dbRDA7** | | | **dbRDA8** | | | **dbRDA9** | | | **dbRDA10** | | | **dbRDA11** | | | **dbRDA12** | | | **dbRDA13** | | | **dbRDA14** | | | **dbRDA15** | | | **dbRDA16** | |  |  |
| **PO1** | -7.0683 | -20.435 | | | 11.748 | | | 9.4898 | | | -6.3321 | | | -5.2494 | | | | 18.641 | | | -0.53753 | | | 7.8621 | | | 3.3351 | | | 20.818 | | | -1.7505 | | | 15.187 | | | -3.0128 | | | 7.3286 | | | 5.6294 | |  |  |
| **PO2** | 2.284 | -20.517 | | | 12.705 | | | 4.9979 | | | -8.0184 | | | 5.4993 | | | | 1.0783 | | | 8.7815 | | | 6.792 | | | 3.1112 | | | -28.024 | | | -1.8004 | | | 8.2463 | | | 4.2902 | | | -9.7489 | | | 3.5607 | |  |  |
| **PO3** | -26.959 | -11.791 | | | -2.0643 | | | -4.2892 | | | -1.5431 | | | -5.5191 | | | | 4.931 | | | -2.8949 | | | 6.1592 | | | 1.5887 | | | -3.2642 | | | -1.199 | | | -21.768 | | | -25.65 | | | -6.3993 | | | -7.8006 | |  |  |
| **PO4** | -20.724 | -13.028 | | | 0.3267 | | | -7.4788 | | | 1.922 | | | 2.2716 | | | | 3.4556 | | | -9.4228 | | | -1.6919 | | | -6.9681 | | | 7.8672 | | | 1.2976 | | | -6.4044 | | | 24.402 | | | -6.0487 | | | -19.587 | |  |  |
| **PO5** | -22.292 | -6.9527 | | | -5.4347 | | | -11.753 | | | 0.1129 | | | 2.6824 | | | | -7.6339 | | | -4.0644 | | | -0.1779 | | | -0.35256 | | | 1.7841 | | | -3.5739 | | | -11.675 | | | 8.3626 | | | 11.845 | | | 28.371 | |  |  |
| **PO6** | -25.643 | 1.0065 | | | -9.2407 | | | 7.0095 | | | -6.4838 | | | 2.0068 | | | | -6.617 | | | 20.109 | | | -23.753 | | | -9.7594 | | | 4.4211 | | | -1.2108 | | | 9.3426 | | | -8.6251 | | | 4.4456 | | | -4.3009 | |  |  |
| **PO7** | -23.827 | 7.4958 | | | -11.25 | | | -18.863 | | | 6.8589 | | | 0.85677 | | | | -4.7019 | | | -14.961 | | | 3.7186 | | | 1.9519 | | | -8.4335 | | | -4.7481 | | | 24.754 | | | -5.3177 | | | -0.76098 | | | -3.1585 | |  |  |
| **PO8** | -8.5742 | 10.331 | | | 0.64067 | | | 0.87045 | | | 4.8591 | | | -0.80546 | | | | 2.5759 | | | 1.6783 | | | -5.6838 | | | 14.48 | | | 5.147 | | | 30.289 | | | 1.3688 | | | 1.3222 | | | -17.518 | | | 8.1848 | |  |  |
| **PO9** | 4.6602 | 27.335 | | | 10.798 | | | -1.2919 | | | -27.089 | | | -7.9395 | | | | 7.9623 | | | -12.684 | | | -6.4862 | | | 1.7159 | | | -1.2441 | | | -6.9599 | | | -2.5062 | | | 1.4993 | | | -4.99E-02 | | | -1.2026 | |  |  |
| **PO10** | 22.562 | 7.0709 | | | -3.0746 | | | -11.65 | | | 21.806 | | | -19.499 | | | | 18.675 | | | 11.808 | | | -2.8577 | | | 2.7889 | | | -2.4379 | | | -9.9615 | | | -0.63323 | | | 1.0603 | | | -0.20175 | | | 1.3049 | |  |  |
| **PO11** | 17.98 | 0.95364 | | | 2.3247 | | | -22.461 | | | -13.807 | | | -0.91168 | | | | -9.2544 | | | 23.434 | | | 8.5557 | | | -2.3835 | | | 4.838 | | | 3.5531 | | | -2.7711 | | | 4.7954 | | | 0.98701 | | | -4.3494 | |  |  |
| **PO12** | 18.105 | 9.7805 | | | 6.2575 | | | -2.4435 | | | 0.47508 | | | 10.25 | | | | 5.9714 | | | -6.6885 | | | 8.6733 | | | -25.841 | | | -2.1239 | | | 11.992 | | | 0.60107 | | | -8.3251 | | | 3.6029 | | | 4.2998 | |  |  |
| **PO13** | 32.994 | -5.8434 | | | -35.374 | | | 6.6926 | | | -7.4769 | | | 14.563 | | | | 7.6429 | | | -3.2266 | | | 4.9981 | | | 5.1972 | | | 2.4948 | | | 0.24601 | | | -1.026 | | | -0.32905 | | | 0.15767 | | | -2.1214 | |  |  |
| **PO14** | 8.9521 | -1.3096 | | | 12.266 | | | 2.5997 | | | 14.411 | | | 17.914 | | | | 8.5115 | | | -2.3974 | | | -18.707 | | | -5.8423 | | | -5.771 | | | -0.46271 | | | -5.4875 | | | 0.92638 | | | 6.0423 | | | -1.4455 | |  |  |
| **PO15** | 26.425 | -4.3827 | | | 12.428 | | | -4.3381 | | | 2.0964 | | | 11.296 | | | | -15.136 | | | -6.9868 | | | -9.2033 | | | 20.027 | | | 8.5187 | | | -10.773 | | | -0.88563 | | | -7.0085 | | | -2.4784 | | | -1.8426 | |  |  |
| **PO16** | -5.9603 | 14.915 | | | 5.9852 | | | 13.45 | | | 12.968 | | | 6.1366 | | | | -13.802 | | | 5.3792 | | | 18.503 | | | -7.3655 | | | 11.662 | | | -11.198 | | | 0.54143 | | | -0.33651 | | | -11.333 | | | 0.23989 | |  |  |
| **PO17** | -12.797 | 11.534 | | | -9.0009 | | | 19.266 | | | -2.0603 | | | -4.2016 | | | | 2.9787 | | | 2.8038 | | | -0.42174 | | | 1.9922 | | | -7.1517 | | | -10.814 | | | -5.752 | | | 9.8116 | | | -6.0945 | | | 5.0959 | |  |  |
| **PO18** | -7.1785 | 8.9069 | | | 2.9525 | | | 10.845 | | | 5.763 | | | -3.4704 | | | | -6.2906 | | | 1.9611 | | | 10.759 | | | 13.507 | | | -7.6898 | | | 11.335 | | | -2.3744 | | | 2.3329 | | | 27.492 | | | -11.731 | |  |  |
| **PO19** | 27.06 | -15.071 | | | -2.9924 | | | 9.3455 | | | 1.5381 | | | -25.88 | | | | -18.988 | | | -12.091 | | | -7.0375 | | | -11.183 | | | -1.4109 | | | 5.739 | | | 1.2431 | | | -0.19801 | | | -1.2672 | | | 0.853 | |  |  |
|  |  | | |  | | |  | | |  | | |  | | |  | | |  | | |  | | |  | | |  | | | |  | | |  | | |  | | |  | | |  | | | |  |
| **B Relationships between dbRDA coordinate axes and orthonormal X variables** | | | | | | | | | | | | | | | | | | | | | |  | | |  | | |  | | | |  | | |  | | |  | | |  | | |  | | | |  |
| **(multiple partial correlations)** | | | | | | |  | | |  | | |  | | |  | | |  | | |  | | |  | | |  | | | |  | | |  | | |  | | |  | | |  | | | |  |
| **Variable** | **dbRDA1** | | **dbRDA2** | | | **dbRDA3** | | | **dbRDA4** | | | **dbRDA5** | | | **dbRDA6** | | **dbRDA7** | | | **dbRDA8** | | | **dbRDA9** | | | **dbRDA10** | | | **dbRDA11** | | **dbRDA12** | | | **dbRDA13** | | | **dbRDA14** | | | **dbRDA15** | | | **dbRDA16** | | |  |  |  |
| **Hg** | 0.453 | | -0.1 | | | -0.339 | | | -0.159 | | | -0.069 | | | -0.085 | | -0.312 | | | 0.264 | | | 0.188 | | | -0.126 | | | 0.246 | | 0.27 | | | 0.047 | | | -0.108 | | | -0.468 | | | 0.219 | | |  |  |  |
| **Cr** | -0.251 | | -0.142 | | | 0.161 | | | -0.003 | | | 0.074 | | | 0.292 | | -0.317 | | | 0.222 | | | 0.115 | | | -0.278 | | | 0.63 | | -0.063 | | | -0.198 | | | 0.09 | | | 0.332 | | | 0.024 | | |  |  |  |
| **Cu** | -0.445 | | 0.255 | | | -0.006 | | | 0.072 | | | 0.031 | | | 0.109 | | -0.278 | | | 0.461 | | | 0.002 | | | 0.248 | | | -0.162 | | -0.387 | | | 0.119 | | | 0.034 | | | -0.405 | | | 0.107 | | |  |  |  |
| **Ni** | -0.121 | | 0.206 | | | -0.319 | | | -0.061 | | | 0.389 | | | -0.002 | | -0.246 | | | -0.579 | | | 0.29 | | | 0.085 | | | 0.022 | | -0.192 | | | -0.281 | | | -0.194 | | | -0.019 | | | 0.212 | | |  |  |  |
| **Pb** | 0.147 | | 0.037 | | | -0.18 | | | 0.216 | | | -0.33 | | | 0.403 | | 0.262 | | | 0.076 | | | 0.384 | | | -0.143 | | | -0.237 | | -0.134 | | | -0.444 | | | 0.32 | | | -0.051 | | | 0.092 | | |  |  |  |
| **As** | 0.156 | | -0.262 | | | 0.252 | | | -0.347 | | | -0.393 | | | 0.077 | | -0.132 | | | -0.295 | | | -0.298 | | | 0.081 | | | 0.05 | | -0.443 | | | -0.032 | | | 0.084 | | | -0.107 | | | 0.381 | | |  |  |  |
| **Cd** | -0.472 | | 0.018 | | | -0.22 | | | -0.494 | | | -0.38 | | | 0.078 | | 0.022 | | | -0.097 | | | 0.259 | | | 0.103 | | | -0.06 | | 0.378 | | | 0.24 | | | 0.117 | | | 0.143 | | | 0.089 | | |  |  |  |
| **Zn** | -0.155 | | -0.123 | | | -0.04 | | | 0.162 | | | -0.251 | | | -0.367 | | 0.071 | | | -0.255 | | | 0.313 | | | -0.196 | | | 0.292 | | -0.321 | | | 0.21 | | | 0.144 | | | -0.304 | | | -0.429 | | |  |  |  |
| **Mn** | 0.087 | | 0.167 | | | -0.146 | | | -0.194 | | | 0.006 | | | 0.252 | | -0.261 | | | -0.154 | | | -0.36 | | | 0.191 | | | 0.074 | | 0.144 | | | -0.237 | | | 0.367 | | | -0.192 | | | -0.575 | | |  |  |  |
| **P** | -0.217 | | -0.022 | | | 0.177 | | | -0.309 | | | 0.233 | | | 0.25 | | 0.236 | | | -0.072 | | | -0.117 | | | -0.628 | | | -0.154 | | 0.062 | | | -0.046 | | | -0.183 | | | -0.416 | | | -0.054 | | |  |  |  |
| **Fe** | 0.089 | | 0.632 | | | -0.049 | | | 0.274 | | | -0.247 | | | 0.281 | | 0.082 | | | -0.206 | | | -0.206 | | | -0.165 | | | 0.297 | | 0.046 | | | 0.356 | | | -0.094 | | | -0.007 | | | 0.175 | | |  |  |  |
| **Al** | -0.123 | | 0.064 | | | -0.019 | | | 0.021 | | | -0.466 | | | -0.073 | | -0.05 | | | 0.08 | | | -0.091 | | | 0.058 | | | 0.019 | | 0.02 | | | -0.425 | | | -0.707 | | | 0.013 | | | -0.234 | | |  |  |  |
| **LOI%** | -0.078 | | -0.514 | | | -0.13 | | | 0.416 | | | -0.045 | | | 0.443 | | -0.308 | | | -0.216 | | | -0.037 | | | 0.01 | | | -0.222 | | 0.11 | | | 0.304 | | | -0.188 | | | -0.039 | | | -0.084 | | |  |  |  |
| **PAH** | 0.184 | | -0.095 | | | -0.429 | | | -0.314 | | | 0.137 | | | 0.295 | | 0.326 | | | 0.184 | | | 0.003 | | | 0.11 | | | 0.132 | | -0.438 | | | 0.253 | | | -0.235 | | | 0.191 | | | -0.227 | | |  |  |  |
| **PCB** | -0.287 | | -0.174 | | | -0.587 | | | 0.176 | | | -0.026 | | | -0.258 | | 0.125 | | | 0.054 | | | -0.52 | | | -0.219 | | | 0.056 | | -0.022 | | | -0.158 | | | 0.167 | | | 0.039 | | | 0.225 | | |  |  |  |
| **silt** | 0.162 | | 0.203 | | | -0.105 | | | -0.13 | | | -0.116 | | | -0.165 | | -0.472 | | | 0.074 | | | 0.005 | | | -0.489 | | | -0.424 | | -0.212 | | | 0.132 | | | 0.042 | | | 0.361 | | | -0.147 | | |  |  |  |

**Supplementary Table 8.** Output of dbRDA correlations. Reported are the coordinates scores for each station in the Mar Piccolo site (A) and the correlations between selected environmental variable, chemical contaminant and dbRDA axis (B).

| **A dbRDA coordinate scores** | | |  |  |  |  |  |  |  |
| --- | --- | --- | --- | --- | --- | --- | --- | --- | --- |
| **Sample** | **dbRDA1** | **dbRDA2** | **dbRDA3** | **dbRDA4** | **dbRDA5** | **dbRDA6** | **dbRDA7** | **dbRDA8** | **dbRDA9** |
| **TA1** | -24 | 6.7087 | 35.782 | 2.4113 | 4.695 | 4.4422 | 1.4537 | -2.7579 | 0.19412 |
| **TA4** | -22.952 | -7.8541 | -15.055 | 0.249 | 0.43442 | 4.6578 | 4.7718 | -6.1229 | 19.511 |
| **TA5** | -26.908 | -12.756 | -14.73 | 6.9508 | 0.62026 | 4.3826 | 7.2216 | 2.5289 | -17.254 |
| **TA7** | 3.9322 | 17.143 | -3.8782 | -29.929 | -2.61E-02 | 8.4835 | -1.8568 | -4.7248 | -4.4216 |
| **TA8** | 9.8578 | 20.782 | -5.6965 | 8.9143 | -12.502 | 16.116 | -1.671 | 10.266 | 1.5512 |
| **TA9** | 16.251 | -19.716 | 1.1974 | -3.5833 | 23.579 | 3.8394 | -2.6047 | 14.691 | 2.6336 |
| **TA10** | 20.106 | -15.003 | -1.6437 | 4.1209 | 3.071 | 0.66253 | -3.3221 | -21.019 | -3.8478 |
| **TA11** | -1.6047 | -19.074 | 4.7252 | -12.474 | -18.089 | -17.956 | 2.5403 | 7.8108 | 1.0374 |
| **TA12** | 20.208 | -11.669 | 8.4918 | 10.109 | -13.413 | 5.133 | -3.4803 | -0.37622 | 0.80357 |
| **TA13** | -9.833 | 17.223 | -7.0714 | 6.7082 | 4.6008 | -15.162 | -23.881 | 0.51905 | -0.5402 |
| **TA14** | 14.942 | 24.215 | -2.1212 | 6.5228 | 7.0308 | -14.599 | 20.828 | -0.81489 | 0.33309 |
|  |  |  |  |  |  |  |  |  |  |
| **B Relationships between dbRDA coordinate axes and orthonormal X variables** | | | | |  |  |  |  |  |
| **(multiple partial correlations)** | |  |  |  |  |  |  |  |  |
| **Variable** | **dbRDA1** | **dbRDA2** | **dbRDA3** | **dbRDA4** | **dbRDA5** | **dbRDA6** | **dbRDA7** | **dbRDA8** | **dbRDA9** |
| **Hg** | -0.518 | -0.326 | -0.428 | 0.516 | 0.205 | 0.023 | 0.205 | -0.125 | 0.275 |
| **Cr** | 0.336 | 0.056 | -0.397 | 0.471 | -0.3 | 0.183 | -0.403 | -0.239 | -0.402 |
| **Pb** | 0.071 | -0.033 | -0.25 | -0.317 | 0.624 | 0.472 | -0.449 | 0 | 0.13 |
| **As** | -0.572 | -0.088 | -0.288 | -0.438 | -0.177 | -0.297 | -0.297 | 0.004 | -0.427 |
| **Zn** | 0.301 | 0.228 | -0.488 | -0.224 | 0.298 | -0.247 | 0.548 | -0.258 | -0.228 |
| **P** | -0.27 | 0.713 | -0.238 | -0.099 | -0.348 | 0.347 | 0.083 | 0.007 | 0.323 |
| **LOI%** | 0.331 | -0.246 | -0.466 | -0.135 | -0.274 | -0.23 | -0.081 | 0.551 | 0.396 |
| **PAH** | -0.035 | 0.497 | 0.038 | 0.315 | 0.362 | -0.59 | -0.37 | 0.15 | 0.114 |
| **PCB** | -0.109 | 0.12 | -0.027 | 0.204 | 0.178 | 0.269 | 0.234 | 0.731 | -0.488 |

Supplementary Figure 1. Principal Coordinates Analysis (PCO) of the environmental variables and chemical contaminants in the two study sites. Green circles: Po River Prodelta samples. Blue circles: Mar Piccolo of Taranto samples.


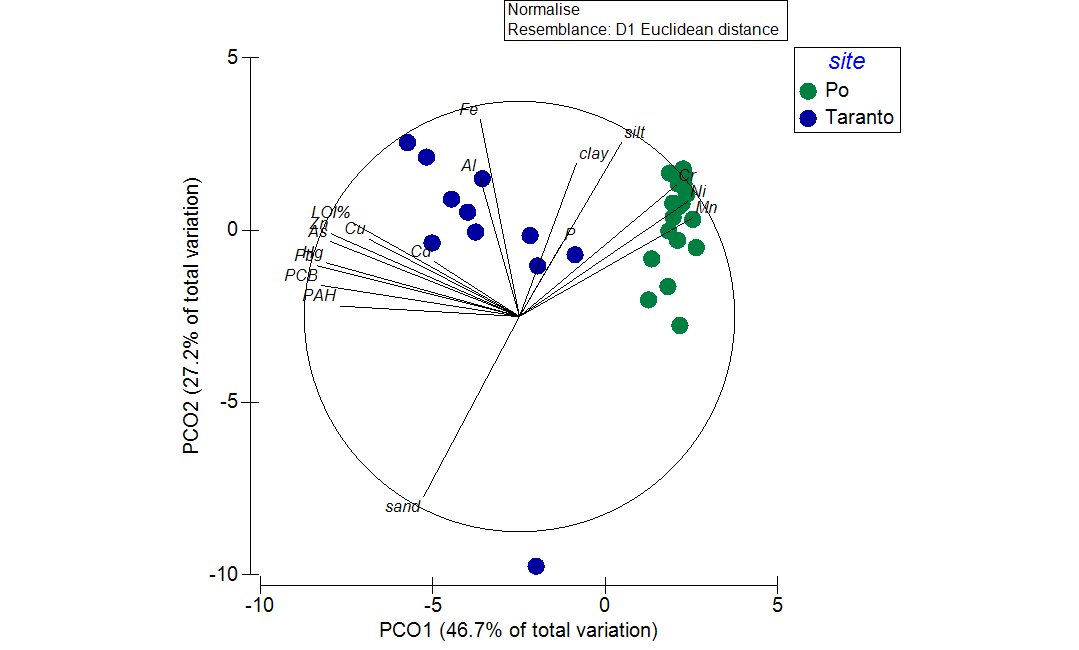


Supplementary Figure 2. OTU richness in the two sites, as resulting from 16S rDNA Illumina sequencing. Green bars refer to the Po River Prodelta site, while blue bars refer to the Mar Piccolo site.


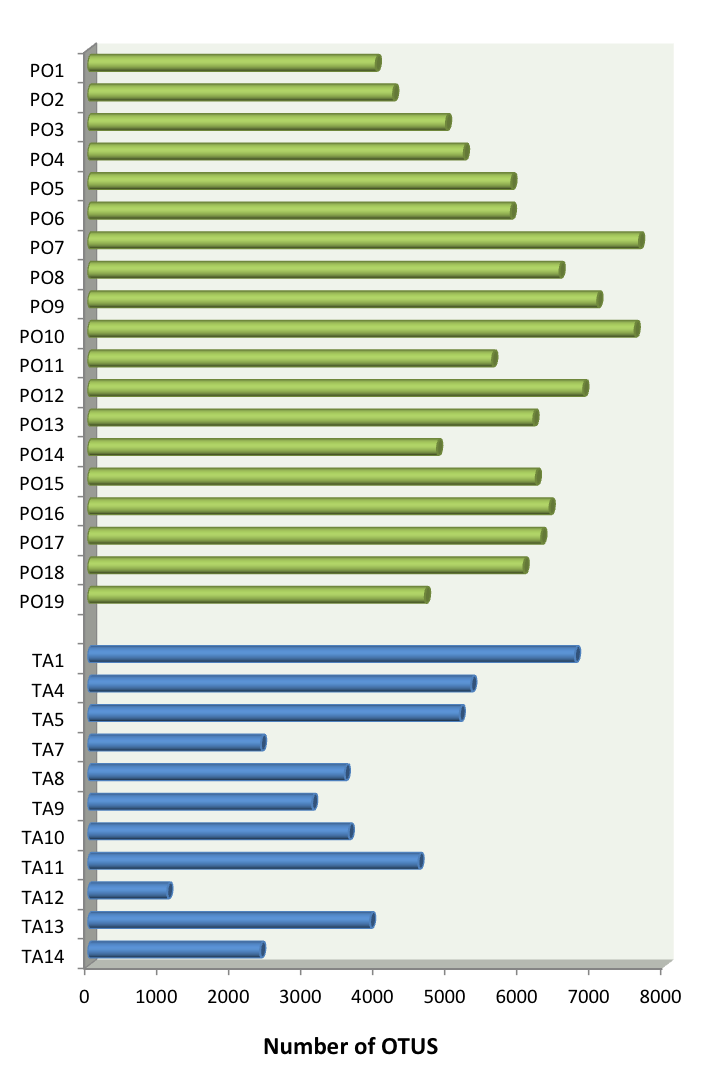

Supplement: Supplementary file 1 [file Data_Sheet_1.DOCX]
